# LolA and LolB from the plant-pathogen *Xanthomonas campestris* forms a stable heterodimeric complex in the absence of lipoprotein

**DOI:** 10.3389/fmicb.2023.1216799

**Published:** 2023-07-12

**Authors:** Valentina Furlanetto, Christina Divne

**Affiliations:** Department of Industrial Biotechnology, School of Engineering Sciences in Chemistry, Biotechnology, and Health (CBH), KTH Royal Institute of Technology, Stockholm, Sweden

**Keywords:** lipoprotein transport, LolA-LolB complex, Gram-negative bacteria, *Xanthomonas campestris*, plant pathogen, crystal structure

## Abstract

The Gram-negative bacterium *Xanthomonas campestris* is one of the most problematic phytopathogens, and especially the pathovar *campestris* (*Xcc*) that causes a devastating plant disease known as black rot and it is of considerable interest to understand the molecular mechanisms that enable virulence and pathogenicity. Gram-negative bacteria depend on lipoproteins (LPs) that serve many important functions including control of cell shape and integrity, biogenesis of the outer membrane (OM) and establishment of transport pathways across the periplasm. The LPs are localized to the OM where they are attached via a lipid anchor by a process known as the localization of lipoprotein (Lol) pathway. Once a lipid anchor has been synthesized on the nascent LP, the Lol pathway is initiated by a membrane-bound ABC transporter that extracts the lipid anchor of the LP from the IM. The ABC extractor presents the extracted LP to the transport protein LolA, which binds the anchor and thereby shields it from the hydrophilic periplasmic milieu. It is assumed that LolA then carries the LP across the periplasm to the OM. At the periplasmic face of the OM, the LP cargo is delivered to LolB, which completes the Lol pathway by inserting the LP anchor in the inner leaflet of the outer membrane. Earlier studies have shown that loss of *Xcc* LolA or LolB leads to decreased virulence and pathogenicity during plant infection, which motivates studies to better understand the Lol system in *Xcc*. In this study, we report the first experimental structure of a complex between LolA and LolB. The crystal structure reveals a stable LolA-LolB complex in the absence of LP. The structural integrity of the LP-free complex is safeguarded by specific protein–protein interactions that do not coincide with interactions predicted to participate in lipid binding. The results allow us to identify structural determinants that enable *Xcc* LolA to dock with LolB and initiate LP transfer.

## Introduction

Outer-membrane (OM) lipoproteins (LPs) perform a wide range of important functions in Gram-negative bacteria such as maintaining cell shape and integrity, as well as acting as virulence factors (El Rayes et al., 2021). While some LPs face the periplasm, others are displayed on the surface and exposed to the surrounding environment (Cole et al., 2021).

**Figure 1:**
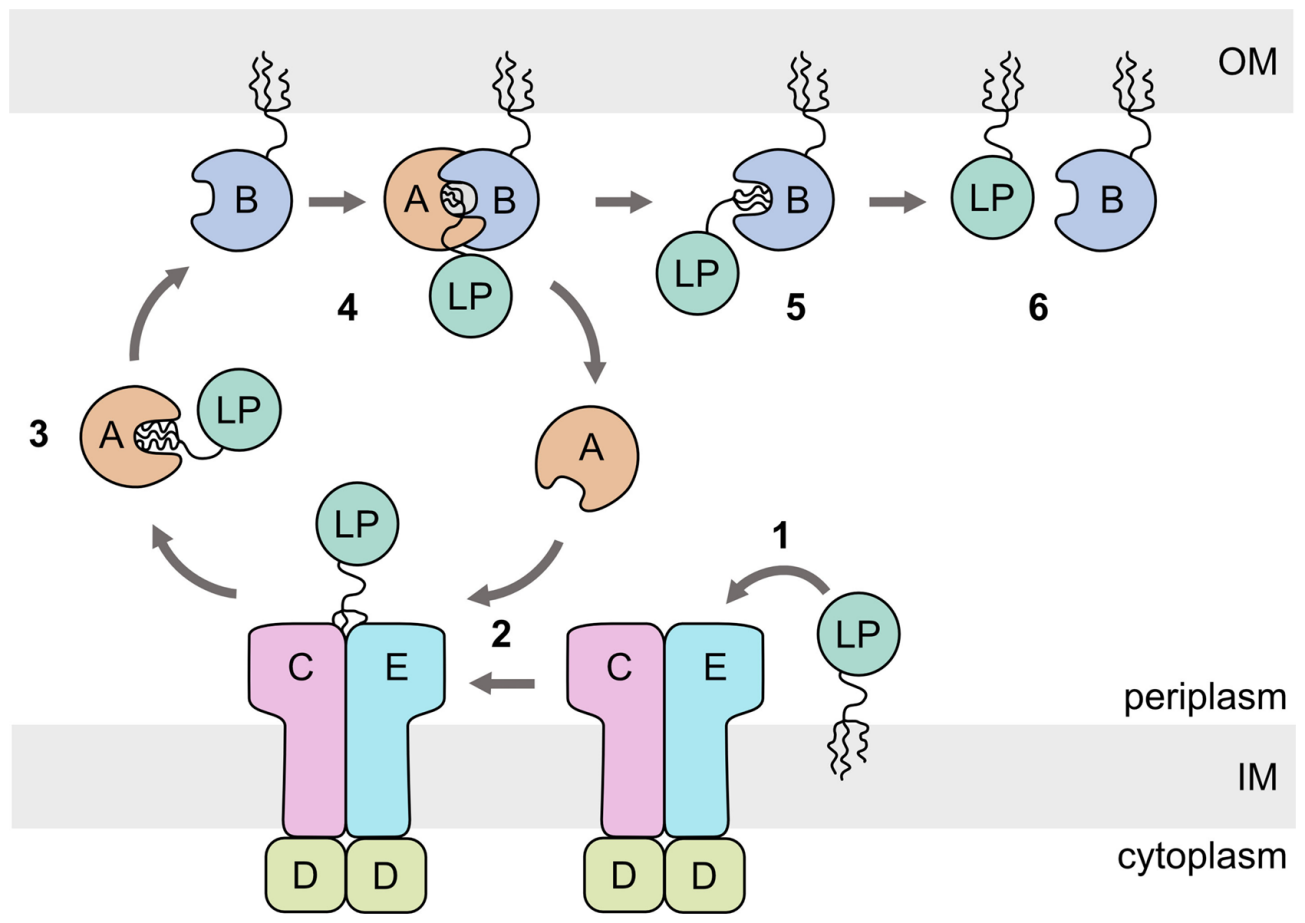
Schematic representation of the *Escherichia coli* lipoprotein outer membrane localization (Lol) pathway. The ABC transporter LolCDE extracts the lipoprotein (LP) from the inner membrane (IM) (1). The lipid anchor is then transferred to LolA (2), which acts as a chaperone for the lipid anchor during crossing of the periplasm (3). LP-bound LolA associates with LolB and transfers the lipid anchor (4,5). LolB inserts the lipid anchor in the OM (6).

The lipoprotein outer membrane localization (Lol) pathway of the Gram-negative bacterium *Escherichia coli* is the best studied system and is considered as the canonical model for how most LPs are transported from the inner to the outer membrane (Figure 1) (Grabowicz, 2018, 2019). The *E. coli* Lol system involves five periplasmic proteins: LolCDE, an ATP-binding cassette (ABC) transporter of type VII (Thomas et al., 2020) that extracts the lipid anchor of the cargo from the inner membrane (Yakushi et al., 1998, 2000; Yasuda et al., 2009; Szewczyk and Collet, 2016); LolA, a soluble protein that receives the lipid anchor from LolCDE and transports the cargo across the periplasm to the outer membrane (Matsuyama et al., 1995); and LolB, a lipoprotein itself, attached to the periplasmic side of the outer membrane that accepts the lipid anchor from LolA and inserts the anchor into the inner leaflet of the outer membrane (Matsuyama et al., 1997).

The lipoprotein membrane anchor attached to LPs synthesized by *E. coli* is a triacylated lipid attached to a protein cysteine residue via a thioester bond to generate N-acyl-*S*-diacyl glyceryl cysteine (Nakayama et al., 2012). The prolipoprotein includes an N-terminal leader sequence (lipoprotein signal peptide) that attaches the prolipoprotein to the inner membrane. Immediately following the signal peptide are four amino acids that constitute a lipobox motif where the fourth residue is a cysteine (Inouye et al., 1977; Okuda and Tokuda, 2011). The cysteine is the attachment site for subsequent synthesis of the lipid anchor. Following the cysteine are three amino acids that determine the subcellular localization of the LP (sorting signals), and a relatively long tether peptide that acts as a spacer between the lipid anchor and the protein (Tokunaga et al., 1982; Gupta et al., 1993; Sankaran and Wu, 1994).

Three enzymes are required for synthesizing triacyl anchor to produce a mature lipoprotein (Supplementary Figure S1): Lgt (phosphatidylglycerol-prolipoprotein diacylglyceryl transferase) attaches a diacylglycerol to the cysteine of the prolipoprotein via a thioester bond to produce a diacylglyceryl prolipoprotein (Sankaran and Wu, 1994); Lsp (lipoprotein signal peptidase; Innis et al., 1984) cleaves off the N-terminal lipoprotein signal sequence to produce an apolipoprotein; and finally, Lnt (apolipoprotein N-acyl transferase) that adds the third acyl chain via an amide bond to the cysteine to generate the mature triacylated hololipoprotein (Gupta et al., 1993). Not all Gram-negative bacteria have Lnt and have been proposed to instead synthesize LPs with diacyl anchors (LoVullo et al., 2015), or other variants (Nakayama et al., 2012), and there are examples of bacteria where Lgt is non-essential (Dautin et al., 2020).

Studies using LolA and LolB knockout mutants (Tanaka et al., 2001; Grabowicz and Silhavy, 2017) have shown that LP trafficking in *E. coli* can bypass the Lol system, and that the most likely biological function of the Lol pathway is to prevent mislocalization and lethal accumulation of LPs in the inner membrane. Whether a functioning Lol system is strictly essential for bacterial survival, and how applicable the *E. coli* model is on Gram-negative bacteria in general is still being investigated (Konovalova and Silhavy, 2015; Grabowicz and Silhavy, 2017; Grabowicz, 2019). Considering the general importance of LP localization and function, such questions are of considerable interest to better understand the Lol system and how it may be targeted for new therapeutics against pathogenic bacteria.

The Gram-negative bacterium *Xanthomonas campestris* is a notorious plant pathogen and the pathovar *campestris* (i.e., *Xanthomonas campestris* pv. *campestris*; *Xcc*) that causes black rot is particularly problematic. Black rot is a devastating plant disease that infects a range of agronomically important cruciferous plants of the genus Brassica, including cabbage, broccoli, and cauliflower (Leyns et al., 1984; Vicente and Holub, 2013). As most Gram-negative bacterial pathogens, the genome of the phytopathogen *Xcc* (Vorhölter et al., 2008) contains genes coding for a tripartite Lol system with an ABC-type extractor, the LolA chaperone and the OM insertase LolB.

The importance of LolA and LolB in *Xcc* has been studied using transposon mutagenesis to generate *lolA*- (Liao et al., 2019) and *lolB*-deficient (Liao et al., 2022) *Xcc* mutant strains. In both cases, the effect of the mutant *Xcc* strains on cabbage was evaluated with similar results: substantial reduction in virulence, reduced pathogenicity, reduced bacterial attachment on abiotic surfaces and host leaves, significantly reduced production of extracellular enzymes, and greatly impaired stress tolerance. Additionally, proteomics analysis showed that the inactivation of LolA or LolB affected the expression of a wide range of additional genes. Furthermore, the mutant phenotypes could be rescued by trans-complementation with the wild-type genes.

In the present study, we present the experimental crystal structure of the LP-free LolA-LolB complex from *Xanthomonas campestris* pv. *campestris*. We show that in solution, LolA and LolB form a heterodimeric, non-obligate protein–protein complex. These findings allow us to offer new insight into the association between LolA LolB in the absence of LP, and to propose interactions that enable lipid binding and transfer.

## Materials and methods

### Cloning of Xcc LolA and LolB

The genes coding for LolA and LolB from *Xcc* strain B100 (LolA, GenBank AM920689, UniProt B0RT42; LolB, GenBank AM920689, UniProt B0RUA2) were synthesized and codon optimized for expression in *E. coli* by Integrated DNA Technologies (IDT, Iowa, United States). The *lolA* and *lolB* gene constructs were cloned using ligation independent cloning (LIC; Doyle, 2005) into the vector pNIC-CTHO (Supplementary Figure S2), which adds a C-terminal hexahistidine tag preceded by a sequence allowing proteolytic cleavage by Tobacco Etch Virus (TEV) protease (Savitsky et al., 2010). The *lolA* gene was cloned without its N-terminal periplasmic signal peptide, and the *lolB* gene without the lipoprotein signal, including the lipobox cysteine where lipidation occurs. The constructs and cloning primers are listed in Supplementary Table S1.

PCR was carried out using 50 ng plasmid DNA and 2 U Phusion High-Fidelity DNA Polymerase (Thermo Fisher) with the addition of 25 μM of the primers, 10 mM dNTP, 1.5 μL DMSO and 5 x HF Phusion buffer (Thermo Fisher). The PCR protocol included four steps: 98°C for 30 s; 30 cycles of 98°C for 10 s, the respective annealing temperature of the designed primers for 60 s, 72°C for 90 s, and a final incubation at 72°C for 10 min. The vector pNIC-CTHO was linearized with the restriction enzymes BveI (Thermo Scientific), at 37°C for 30 min. DpnI-digested PCR products and digested LIC vector were purified with PCR Purification Kit (Qiagen) and treated with T4 DNA polymerase to generate overhangs. Inserts and vectors were incubated for 10 min at room temperature and transformed into *E. coli* DH5α cells (Invitrogen) grown on Luria-Bertani (LB) agar supplemented with 50 μg ml^−1^ kanamycin (37°C, 12 h).

### Production and purification of LolA and LolB

The commercially available Mix&Go! Transformation Kit (Zymo Research, Nordic Biosite) was used to make *E. coli* BL21(DE3)-T1 cells competent, followed by separate transformations of the recombinant plasmids carrying *lolA* and *lolB* into competent *E. coli* BL21(DE3)-T1 competent cells. The cells were grown at 37°C in 2 L Terrific Broth (TB) containing 50 μg ml^−1^ kanamycin. At an OD_600_ of 0.6, the temperature was lowered to 18°C, and at an OD_600_ of 1.0 the gene expression was induced by adding *β*-D-1-thiogalactopyranoside (IPTG) to a final concentration of 0.1 mM. The cells were harvested after 16 h by centrifugation (5,000 × g, Sorvall BIOS 8 floor centrifuge, Thermo Fisher Scientific).

The resulting bacterial pellets were resuspended separately in 20 mM tris(hydroxymethyl) aminomethane hydrochloride (Tris–HCl) buffer pH 7.8, 150 mM NaCl, 5% (v/v) glycerol, and one tablet of cOmplete™ Protease Inhibitor Cocktail (Roche). Each resuspended pellet was homogenized using an AVESTIN Emulsiflex-C3 system (Avestin Europe, GmbH), followed by sonication (1 min, 40% intensity, pulse on/off 1.5 s). The lysate was centrifuged using an Avanti J-20XP centrifuge (Beckman Coulter) at 10,000 r.p.m. (Beckman Coulter JA-25.50 fixed-angle rotor, 12,096 Å × g) for 10 min at 4°C, and the resulting supernatant containing target protein was recovered.

For protein capture, immobilized nickel-affinity chromatography (Ni^2+^-IMAC) was performed. The supernatant was mixed with Ni-NTA agarose resin (Invitrogen), left to incubate for 1 h, and packed in Bio-Rad Econo-Pac® columns (Bio-Rad Laboratories, Inc). The columns were washed with 20 mM TRIS–HCl pH 7.8, 150 mM NaCl, 5% (v/v) glycerol, and 10–50 mM imidazole (10 mM of imidazole in the equilibration buffer, 30 mM of imidazole in the first wash step, and with 50 mM of imidazole in the second wash step).

Bound protein was eluted with the same buffer but with 500 mM imidazole and concentrated using Pierce™ Protein Concentrator PES (Thermo Scientific; molecular weight cut-off, MWCO, 10 kDa), and loaded onto a HiLoad 16/60 Superdex 200 prep grade column (GE Healthcare Life Sciences) equilibrated with 20 mM TRIS–HCl pH 7.8, 150 mM NaCl, 5% (v/v) glycerol. The recovered protein-containing fractions were pooled and concentrated to final concentration ranging from 50 to 80 mg ml^−1^ (Pierce™ Protein Concentrator PES; MWCO 10 kDa).

### Size-exclusion chromatographic (SEC) analysis

LolA and LolB were mixed in a molar ratio 1:1 and incubated at room temperature for 1 h. The sample was loaded onto a HiLoad 16/60 Superdex 200 prep grade column (GE Healthcare Life Sciences) equilibrated with with 20 mM TRIS–HCl pH 7.8, 150 mM NaCl, and 5% (v/v) glycerol. The fractions containing LolA-LolB heterodimers were pooled and concentrated to 40–50 OD_280_ ml^−1^ (Pierce™ Protein Concentrator PES; MWCO 10 kDa).

### Isothermal titration calorimetry (ITC)

The ITC experiments were performed with a Microcal iTC200 (Malvern Panalytical) at the thermostatic temperature of 25.0°C, reference power of 6.00 μcal s^−1^, stirring speed 1,000 r.p.m., initial delay 60 s, the first injection of 1 μL followed by 15 injections of 2.5 μL. The proteins were dialyzed against the same batch of buffer composed of 20 mM TRIS–HCl (pH 7.8), 150 mM NaCl, 5% glycerol (v/v). In all experiments, controls were used to isolate the enthalpic contribution corresponding to only the binding of the studied interaction partners.

The concentration of the macromolecule in the cell ([M]_t_) was calculated by estimating the expected dissociation constant (*K*_d_) and applying the formula for the Wiseman *c* value, where *c* = *n*[M]_t_/*K*_d_, *n* is the number of binding sites per macromolecule M (Wiseman et al., 1989); and the *c* value typically fall in the range 3 to 100 (optimally around 30). The concentration in the syringe was set to be 10 to 20 times higher than the concentration in the cell. Estimating a *K*_d_ of 10 μM and a *n* = 1, LolA was concentrated to 1 mM and loaded in the syringe, while LolB was diluted to 0.1 and loaded in the cell. The data were processed using Microcal PEAQ-ITC analysis software supplied by the manufacturer. After the heat of dilution was subtracted using controls, the raw data were fitted considering a 1:1 binding model, floating all the variable parameters, such as binding enthalpy, affinity constant and number of sites.

### Crystal-structure determination and analysis

*Xcc* LolA and LolB were incubated in 20 mM Tris–HCl (pH 7.8), 150 mM NaCl, 5% glycerol (v/v), and subjected to SEC (HiLoad 16/60 Superdex 200 prep grade column) (GE Healthcare Life Sciences) equilibrated with the same buffer to remove free LolA and LolB. Crystallization of the LolA-LolB complex was performed using the vapor diffusion method in sitting drops at room temperature. Several crystallization screens were tested but only MemGold™ (Molecular Dimensions) proved successful.

Prior to data collection, 10% glycerol was added to the drop containing the crystals, the crystals harvested, and vitrified in liquid nitrogen. X-ray intensity data were collected on several crystals using synchrotron radiation at BioMAX (MAX IV, Lund, Sweden) under cryogenic conditions (100 K). The *XDS* package was used for processing and scaling (Kabsch, 2010). The LolA-LolB complex was found to crystallize in space group R3 (no. 146) with varying degree of twinning ranging from severe to mild. Screening of many data sets allowed the identification of an untwinned R3 crystal grown from a mixture of 0.1 μL protein solution (43 OD_280_ ml^−1^ LolA and LolB in 20 mM Tris–HCl (pH 7.8), 150 mM NaCl, 5% glycerol (v/v)) and 0.2 μL protein reservoir containing 50 mM Hepes (pH 7.5), and 2.5 M (NH_4_)_2_SO_4_. The cell dimensions in the hexagonal setting were *a* = *b* = 137.51 Å, *c* = 145.21 Å, α = β = 90°, γ = 120° with four predicted molecules in the asymmetric unit corresponding to a solvent content of approximately 56%. The data were processed and scaled to 2.2 Å resolution (Supplementary Table S2).

Phasing was performed using molecular replacement with *PHENIX Phaser* (Adams et al., 2010). Individual homology models of LolA and LolB to be used as search models were prepared using AlphaFold2 (Jumper et al., 2021) with MMseq2 as implemented in the Google Colaboratory resource (ColabFold; AlphaFold2.ipynb; Mirdita et al., 2022). Loops and regions expected to be flexible were removed from the LolA and LolB search models. Molecular-replacement phasing was performed in steps. The first solution included one LolA and was relatively weak, but nonetheless showed a crystal packing likely to accommodate several additional molecules. Fixing the first LolA molecule and searching for a second failed. Instead, fixing the first LolA molecule and searching for one LolB molecule returned a reasonable solution for a complete LolA-LolB complex. Further fixing this solution and searching for a second LolA-LolB complex returned a strong solution with log likelihood gain (LLG) of 793.028 and a final translation function Z-score (TFZ) of 27.5.

The initial solution was refined using *phenix.refine* (Adams et al., 2010) and included reciprocal-space refinement of *x*,*y*,*z* coordinates, individual *B*-factor refinement and TLS (Translation-Libration-Screw-rotation model). During this first refinement round, the *R* and *R*_free_ values decreased from 0.4757 to 0.2838 and 0.4606 to 0.3340, respectively. The resulting map coefficients for σ_A_-weighted 2*F*_o_-*F*_c_ and *F*_o_-*F*_c_ electron-density maps allowed continued iterative manual correction of the model using *COOT* (Emsley et al., 2010) and refinement with *PHENIX*. Statistics for the final model refined at 2.2 Å resolution is provided in Supplementary Table S2. The oligomeric state present of the refined *Xcc* LolA-LolB complex was analyzed using *PDBePISA* (Proteins, Interfaces, Structures and Assemblies; https://www.ebi.ac.uk/pdbe/pisa/; Krissinel and Henrick, 2007) to evaluate the structural and chemical properties of the interfaces, and the biological significance of the protein–protein interactions (PPIs).

### Modeling of lipoprotein diacyl anchors in LolA and LolB

To evaluate the conformational states representing LP-binding modes before and after transfer of a lipoprotein from LolA to LolB, the diacyl-bound states of LolA and LolB were modeled based on the experimental structure of the LolA-LolB complex. The triacyl anchor fitted in the experimental electron density of the lipid-bound *E. coli* LolA crystal structure (PDB 7Z6W; Kaplan et al., 2022) was used to generate a diacyl anchor by removing the third acyl chain (R3), leaving only the R1 and R2 acyl chains attached at the thiol side of the original triacyl anchor. The diacyl anchor was modeled in the hydrophobic cavities of LolA and LolB separately. The only residues that were allowed to move were those not directly engaged in critical LolA-LolB interface interactions. The varying conformational states of experimental *E. coli* LolA structures currently available in the Protein Data Bank (2ZPC and 2ZPD, Oguchi et al., 2008; 6F3Z, Kaplan et al., 2018; 7Z6W, Kaplan et al., 2022) were used to extrapolate a likely conformation for the lipid-bound state of LolA in the LolA-LolB complex. All model manipulation was performed manually using *COOT* (Emsley et al., 2010).

### Comparative structural analysis

Protein sequences for a selection of pathogenic Gram-negative bacteria were retrieved from UniProt.^1^ The sequences were analyzed for signal peptides, either a periplasmic target peptide (Sec/SPI; LolA) or a lipoprotein signal peptide (Sec/SPII; LolB) using SignalP-6.0 (https://services.healthtech.dtu.dk/services/SignalP-6.0/; Teufel et al., 2022). The retrieved amino-acid sequences were used to first calculate multiple-sequence alignments (MSAs) using Clustal Omega (https://www.ebi.ac.uk/Tools/msa/clustalo/; Sievers et al., 2011), after which the MSAs were adjusted and corrected manually to agree with structural superpositions of the experimental models available for LolA and LolB: *Xcc* LolA (this work), *E. coli* LolA (1IWL and 1U8A; Takeda et al., 2003), *Pseudomonas aeruginosa* LolA (2W7Q; Remans et al., 2010) and *Yersinia pestis* LolA (4KI3; unpublished); *Xcc* LolB (this work) and *E. coli* LolB (1IWM; Takeda et al., 2003). The final MSAs for LolA and LolB were visualized using ESPript3.0 (https://espript.ibcp.fr/ESPript/ESPript/; Robert and Gouet, 2014). The root-mean-square (r.m.s.d) values were derived by pairwise superposition of the experimental models using the *SSM Superpose* command in *COOT* (Emsley et al., 2010).

## Results

### Structure of *Xcc* LolA and LolB

**Figure 2:**
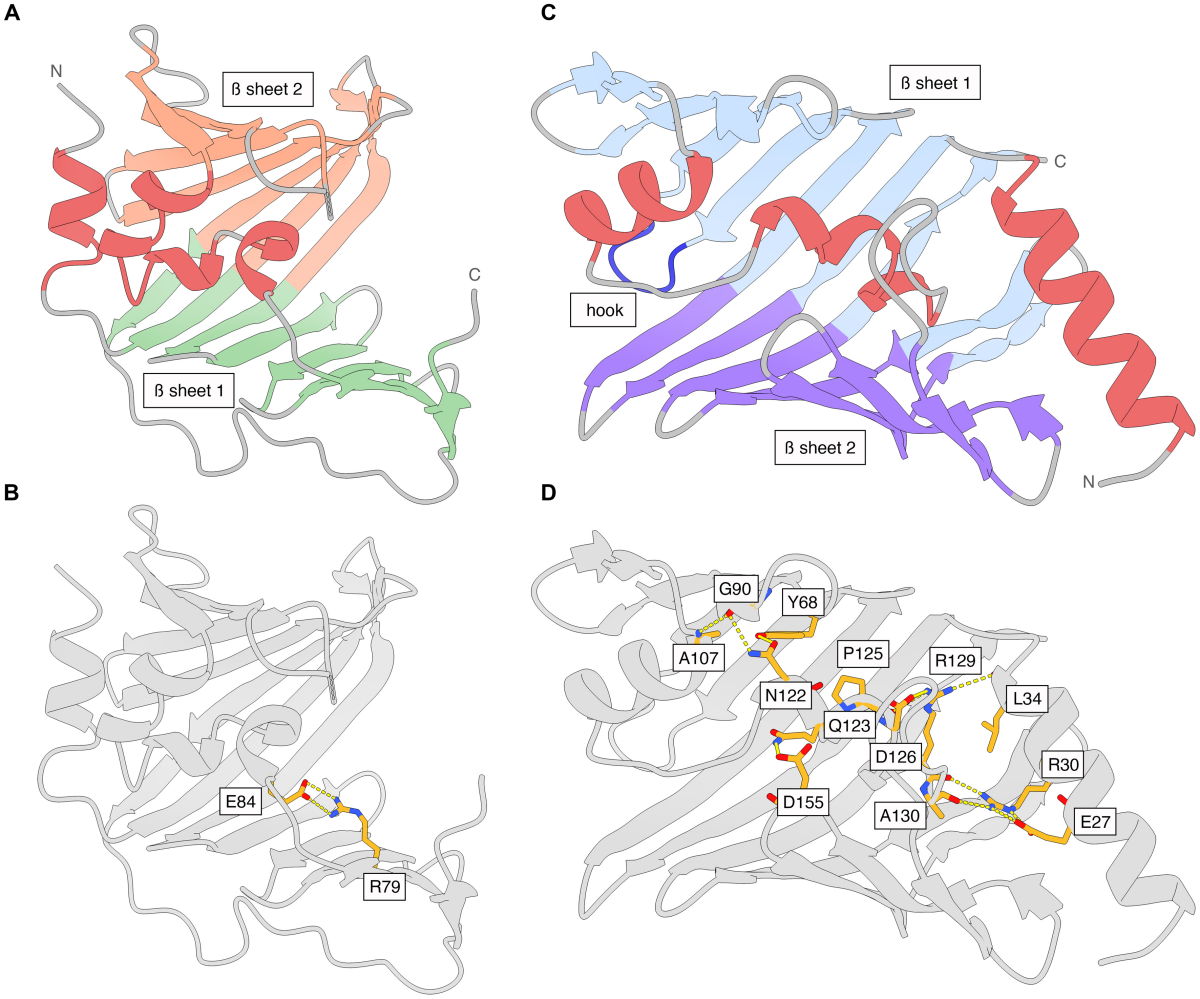
The structures of *Xcc* LolA and LolB shown separately and with interactions that stabilize the helical lids. **(A)** Ribbon representation of *Xcc* LolA and **(B)** LolB. The color scheme is the same as that used in the topology diagrams in Supplementary Figure S3. **(C)** Key interaction stabilizing the helical lid in *Xcc* LolA. Additional interactions are expected to stabilize the lid in LolA but due to the overall weak definition of side chains in LolA, only the Arg79-Glu84 pair is shown. **(D)** Key interactions stabilizing the helical lid in *Xcc* LolB. The figure was prepared by ChimeraX (Pettersen et al., 2021).

The N-terminal periplasmic signal sequence in LolA includes residues 1–21 (UniProt B0RT42) and the mature protein starts at Gly22 in the translated genome sequence, which is numbered Gly1 in the PDB file (Supplementary Table S1). LolA has a β-barrel-like fold with a 11-stranded antiparallel β-sheet (β1A-β11A) and an additional C-terminal β-strand (β12A) that is parallel with β11A (Figure 2A; Supplementary Figure S3). There are four α-helices (α1A-α4A), of which three form a small α-helical domain (α2A-α4A; residues 84–103) that folds as a lid over the hydrophobic, concave inner face of β-sheet 1 to create a hydrophobic cavity. The backbone of the lid is traceable in the electron density while several side chains are less well defined due to flexibility. The position of the helical lid in LolA is mainly stabilized by a salt bridge between Arg79 Nh2 (end of β6A) and Glu84 Oe2 (α2A; Figure 2B). The loop connecting β8A and β9A (residues 122–130) lacks electron density due to high flexibility in LolA of both heterodimers. Additionally, weak electron density is observed for residue 87 in α1A of LolA^1^ (chain A), and 86–87 in LolA^2^ (chain C). LolA chains A and C could both be traced in the electron density to the last residue Gln188.

The lolB gene construct was truncated to omit the N-terminal lipoprotein signal sequence including the lipobox (residues 1–20 in B0RUA2) and the cysteine to be lipidated (residue 21 in B0RUA2), and hence, the first amino acid of recombinant *Xcc* LolB is Val2 (Supplementary Table S1). The region following the cysteine includes the Lol sorting signals (residues 2–4) and a flexible tether region that allows distancing of the protein and lipid part of the lipoprotein cargo to avoid steric clashes. In *Xcc* LolB, the tether comprises residues 5–17 and is too flexible to provide interpretable electron density, and the first visible residue is Val19 in both LolB molecules (chains B and D). The two LolB chains could be traced in the electron density to the last residue (Pro198). The C-terminal proline residue of the two LolB molecules lie at a non-crystallographic two-fold symmetry axis.

Like LolA, LolB displays an antiparallel β-barrel, but with 11 β-strands (β1B-β11B) rather than 12. The α-helices α2B-α3B pack against the concave β-sheet to enclose a hydrophobic cavity (Figure 2C; Supplementary Figure S3). While α2B and α3B form a lid-like structure akin to that seen in LolA, the precise arrangement of the α-helices is different. A six-residue long loop between β3B and β4B in LolB (residues 74–79) forms a hook that inserts like a wedge between the two β-sheets in LolA (Figure 2C).

Besides many hydrophobic interactions, the position of the lid in LolB is secured in the present orientation and conformation by several salt bridges and hydrogen bonds where Arg30 appears to play a key role: Arg30 Nh1 (α1B)–Arg129 O (α3B); Arg30 Nh2 (α1B)–Glu27 Oe2 (α1B); Arg30 Ne (α1B)–Glu27 Oe1 (α1B); Arg30 Nh2 (α1B)–Ala130 O (α3B); Gln123 Ne2 (α3B)–Asp155 Od1 (β8B/β9B); Ala107 N (α2B)–Gly90 O (β5B); Asn122 Nd2 (α3B)–Gly90 O (β5B); Arg129 Nh1 (α3B)–Leu34 O (α1B); Arg129 Nh2 (α3B)–Asp126 Od1 (α3B); Arg129 Nh2 (α3B)–Pro125 O (α3B); Asn122 Od1 (α3B)-Tyr68 OH (β3B) (Figure 2D).

### The *Xcc* LolA-LolB complex

**Figure 3:**
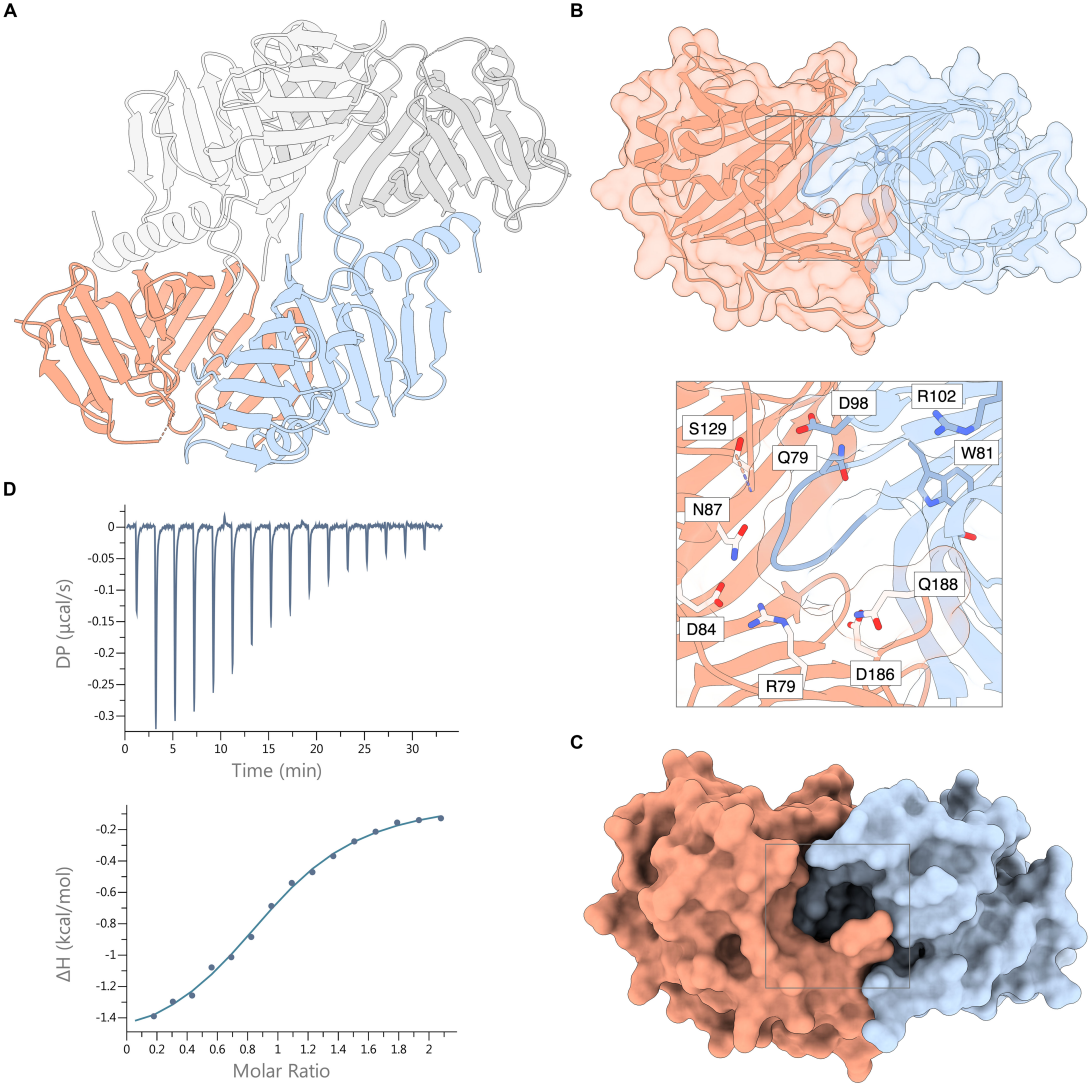
Crystal structure of the *Xcc* LolA-LolB complex and ITC thermogram of their interaction. **(A)** The asymmetric unit of the crystal consists of two LolA-LolB heterodimers 1 and 2, here shown as ribbon representations. Heterodimer 1 includes LolA (chain A, orange) and LolB (chain B, light blue); and heterodimer 2 consists of LolA (chain C, dark gray) and LolB (chain D, light gray). **(B)** Ribbon representation of the *Xcc* LolA-LolB complex overlaid with a semitransparent molecular surface (LolA in orange, LolB in light blue). The LolB hook and Trp81 immediately after the hook are colored in darker blue. The gray box outlines the region of the annulus lined with polar residues. The inset shows a zoom-view of the annular opening and the polar residues lining the annulus. Ser83 is also positioned at the annulus but is not shown due to its ambiguous side-chain conformation. **(C)** The same surface representation as in (B) but with an opaque molecular surface to highlight the annular opening of the LP-free closed LolA-LolB complex. **(D)** ITC thermogram (top) and titration curve (bottom) of association of *Xcc* LolA and LolB. The figure was prepared by ChimeraX (Pettersen et al., 2021).

SEC analysis showed a single, monodisperse peak corresponding to a 1:1 complex of *Xcc* LolA and LolB (Supplementary Figure S4). The crystal structure of the *Xcc* LolA-LolB complex was determined in the trigonal crystal form R3 with two LolA-LolB heterodimers in the asymmetric unit. LolA-LolB heterodimer 1 (LolA^1^-LolB^1^) consists of chains A and B, and LolA-LolB heterodimer 2 (LolA^2^-LolB^2^) of chains C and D (Figure 3A).

In both heterodimers, LolA and LolB interact in a “mouth-to-mouth” orientation with the LolB hook inserted between the two β-sheets of LolA (Figures 3A,B), similar to what was first proposed by Okuda and Tokuda (Okuda and Tokuda, 2009). An annular opening is formed on one face of the LolA-LolB complex (Figures 3B,C). The annulus opens to the solvent where the tether of a bound lipoprotein would protrude and is lined with polar residues: Arg79, Ser83, Glu84, Asn87, Asp127, Ser129, Asp186 and Gln188 from LolA; and Gln79, Asp98, Arg102 and Thr115 from LolB. Immediately below the protein surface, the annulus forks into two paths leading into the lipid-binding hydrophobic cavities in LolA and LolB, respectively. In this lipid-free LolA-LolB complex, the LolB hook is positioned at the bottom of the annulus (Figure 3B and inset).

The thermodynamic properties of association of LolA and LolB were estimated by triplicate ITC experiments where data were fitted using a 1:1-binding model with an *n*-value of 0.96 ± 0.04 and a *K*_d_ value of 14.6 ± 1.0 μM (Figure 3D). The association is exothermic (releases heat) with a Gibbs free energy change (ΔG) of −6.60 ± 0.04 kcal mol^−1^ and is mostly entropy-driven (-TΔS, −4.97 ± 0.01 kcal mol^−1^) with a small but favorable enthalpic (ΔH) contribution of −1.63 ± 0.03 kcal mol^−1^.

**Figure 4:**
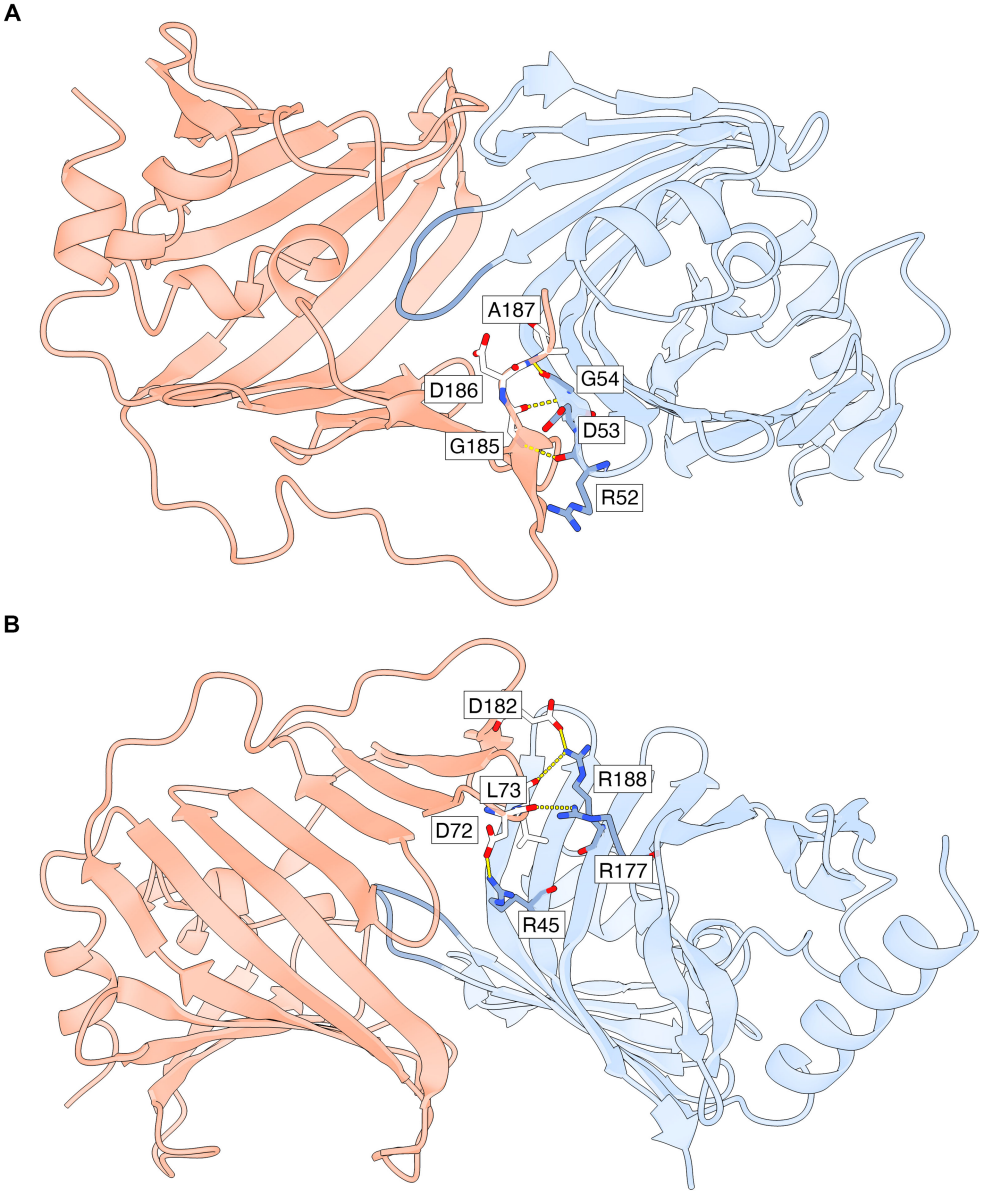
Specific interactions that stabilize the LP-free LolA-LolB complex. Only AB-specific hydrogen bonds and salt bridges that are formed for side chain and backbone atoms with well defined electron density are shown. The interface is also stabilized by additional interactions that are not shown (polar, van der Waals and hydrophobic interactions). LolA and LolB are shown as ribbon representations with the same coloring scheme as in Figure 3, including the LolB hook in darker blue. **(A)** Parallel β-backbone hydrogen bonds between strand β12A in LolA β-sheet 1 and strand β2B in LolB β-sheet 2. **(B)** Rotated view relative to **(A)** to show side chain-mediated hydrogen bonds and salt bridges in LolA and LolB. This view also shows clearly how the inner face of LolA β-sheet 1 interacts with the outer face of LolB β-sheet 2. The figure was prepared by ChimeraX (Pettersen et al., 2021).

Hydrogen bonds and salt bridges that stabilize the lipid-free LolA-LolB complex in both heterodimers of the asymmetric unit (Figure 4; Supplementary Table S3) include: parallel β-type backbone hydrogen bonding between β12A (residues 185–187) in LolA β-sheet 1 and β2B (residues 52–54) in LolB β-sheet 2; hydrogen bonds between LolA Asp72 backbone and LolB Arg177 side chain; hydrogen bond between LolA Leu73 backbone and LolB Arg188 side chain; and possible salt bridges between the side chains of LolA Asp72 and LolB Arg45; and the side chains of LolA Asp182 and LolB Arg188.

The LolB hook (residues 74–79 in chains B and D) assumes slightly different conformations in the two heterodimers to allow some conformational freedom when docked to LolA in the absence of bound lipid (Supplementary Figure S5A). Thus, the hook residues do not participate in interactions that stabilize the LP-free LolA-LolB complex. As an example, the hook residue Arg78 has different conformations in the two LolB molecules but very weak electron density, indicating high conformational freedom. Due to the different conformation of the LolB hook in the two non-crystallographically related heterodimers, LolA and LolB in heterodimer 1 (chains A and B) are slightly more tightly associated than LolA and LolB in heterodimer 2 (chains C and D). This is because the conformation of the LolB hook in heterodimer 1 allows the β-strands of β-sheet 2 in LolA to come closer to LolB.

In the lipid-free LolA-LolB complex, both paths that lead from the annulus to the hydrophobic cavities in LolA and LolB are closed off. The path leading to the LolA cavity is blocked by residues in the LolA lid domain and the LolB hook, and the path to the LolB cavity is blocked by Trp81 in LolB. The closed hydrophobic cavity in LolB appears somewhat tighter than that in LolA, resembling a channel rather than a cavity.

### *PDBePISA* interface analysis

The two heterodimers in the asymmetric unit of the crystal are related by two-fold non-crystallographic symmetry to form a homodimer of heterodimers. The LolA and LolB molecules in the crystal structure interact to generate five unique protein–protein interfaces: LolA^1^-LolB^1^, LolA^1^-LolB^2^, LolA^2^-LolB^2^, LolA^2^-LolB^1^ and LolB^1^-LolB^2^. *PDBePISA* was used to evaluate the stability and possible biological relevance of the interfaces.

The LolA-LolB heterodimer has association type AB and heterodimer 1 was estimated to be stable in solution with complexation significance score (CSS) of 1.0 indicating a biologically relevant association. The interface area of the AB complex (heterodimer 1) was 1,302 Å^2^, a solvation free energy gain (ΔG^int^) upon assembly of −6.1 kcal mol^−1^. As mentioned above, the association of LolA and LolB in heterodimer 2 is less optimized compared to heterodimer 2, which is further emphasized by a smaller calculated interface area of 1,140 Å^2^ and a ΔG^int^ value of −1.7 kcal mol^−1^.

### Modeling lipid binding in *Xcc* LolA and LolB

The hydrophobic residues that form the hydrophobic cavity in LolA are distributed throughout the sequence with the exception for a few regions (Supplementary Table S4). A total of 33 hydrophobic side chains are pointing into the cavity in LolA. As for LolA, hydrophobic residues in LolB that are available for lipid binding are dispersed throughout the sequence. We predict that 41 side chains are suitably positioned to provide hydrophobic interactions with a bound lipid anchor (Supplementary Table S4). Additionally, Pro75 and Val76 in the LolB hook region provide hydrophobic interactions inside LolA.

We analyzed the genome of *Xcc* strain B100 and could not identify any gene corresponding to the enzyme responsible for adding the third acyl chain to a lipoprotein anchor (apolipoprotein N-acyltransferase, Lnt). The absence of Lnt would result in lipoproteins carrying diacylated anchors (*S*-diacyl glyceryl cysteine) as opposed to the triacylated counterpart (*N*-acyl-*S*-diacyl glyceryl cysteine) used by the model bacterium *E. coli*. We therefore modeled a diacyl anchor to either LolA or LolB to evaluate the possible binding modes for pre- and post-lipid transfer.

To model a diacyl lipid in the LolA cavity, it is sufficient to move three regions: the region including the LolA helical lid (residues 82–102; α2A-α4A); the flexible loop connecting β8A and β9A (residues 123–130); and a slight shift of the LolB hook toward the inner β-sheet 2 in LolA. Fitting a diacyl lipid in the LolB cavity is straightforward, only the helical lid (α2B-α3B; residues 105–136) in LolB needs to be displaced. This movement is facilitated by Gly104 and Gly137 on either side of the lid that appear to function as hinges to allow opening of the lid. An additional glycine hinge (Gly116) is present between α2B and α3B that would enable α2B to adjust its position separately when needed. The lid in LolB appears strikingly well designed with its extensive coverage of the cavity and the strategically positioned hinges to control accessibility to the hydrophobic cavity. Based on these observations it was straightforward move the lids to allow positioning of the diacyl anchors in LolA and LolB.

As modeled in LolA, the two ester-bound acyl chains of the diacyl anchor have different opportunities for hydrophobic interactions where the R1 (*sn*-1)- and R2 (*sn*-2)-linked acyl chain can interact with six and four hydrophobic side chains, respectively (Supplementary Table S4). In LolB, the two acyl chains are provided with more hydrophobic contacts, eight for each chain (Supplementary Table S4). The anchor used for modeling was from the crystal structure of lipid-bound LolA from *E. coli* (PDB 7Z6W; Kaplan et al., 2022) which has two saturated palmitoyl chains (C16:0) at the R1 and R2 positions. The length of fatty acids attached at these positions are typically C16 or C18 (Nakayama et al., 2012), and we cannot exclude the possibility that the acyl-chain lengths used by *Xcc* is longer than C16 by one or two carbon atoms, which we predict would be possible to accommodate in *Xcc* LolA and LolB.

### Comparative structural analysis

A selection of LolA and LolB amino-acid sequences from predominantly pathogenic Gram-negative bacteria were aligned and adjusted to agree with structural superposition of available experimental crystal structures (Supplementary Figure S6). The three major classes of the major phylum of Gram-negative bacteria were represented: Gammaproteobacteria including *Xanthomonas campestris* pv. *campestris*, *Xylella fastidiosa*, *Legionella pneumophila*, *Pseudomonas syringae*, *Pseudomonas aeruginosa*, *Yersinia pestis*, and *Escherichia coli*; Betaproteobacteria including *Neisseria meningitidis* and *Neisseria gonorrhoeae*; and Alphaproteobacteria including *Caulobacter vibrioides* and *Rickettsia prowazekii*.

Based on the percent sequence identity for pairwise aligned sequences between *Xcc* LolA and the bacterial homologs (Supplementary Table S5) only *X. fastidiosa* LolA is closely related to *Xcc* LolA (65% sequence identity). The second most similar group includes *Pseudomonas*, *Legionella* and *Neisseria* (32–37% sequence identity). The LolA homologs with low sequence identity include *E. coli*, *Y. pestis*, *C. vibrioides* and *R. prowazekii* (18–24% sequence identity). Except for the most similar LolB from *X. fastidiosa* LolB (51% sequence identity), the LolB homologs show a different pattern with the two *Pseudomonas* homologs being the second most similar group (28–30% sequence identity), and all other homologs displaying low sequence identities to *Xcc* LolB of 18 to 22%.

Experimental structures are available for proteins that resemble the LolA/LolB fold, but only a few have been confirmed as true homologs. We compared *Xcc* LolA with three unliganded wild-type LolA structures, namely the original LolA structure from *E. coli* (1IWL and 1UA8; Takeda et al., 2003), *P. aeruginosa* LolA (2W7Q; Remans et al., 2010), and the deposited but unpublished structure of *Y. pestis* LolA (4KI3). The r.m.s.d. values for pairwise structural superpositioning were: *Xcc* LolA-1IWL, 2.4 Å (147 aligned residues); *Xcc* LolA-2W7Q, 2.5 Å (161 aligned residues); and *Xcc* LolA-4KI3, 2.0 Å (156 aligned residues). For LolB, only the crystal structure of *E. coli* is available (1IWM; Takeda et al., 2003). Pairwise aligned r.m.s.d for the pair *Xcc* LolB–1IWM was 2.1 Å (163 aligned residues).

*E. coli* is the only other bacterium for which experimental structures are available for both LolA and LolB, and where mutagenesis experiments have been performed to investigate LP binding and transfer. Unfortunately, due to the low sequence identity (Supplementary Table S5), direct comparison between *Xcc* LolA and LolB and the *E. coli* counterparts proved difficult. The crystal structures for *V. cholerae* LolA and LolB were recently determined (Jaiman et al., 2023) but have not yet been made publicly available by the Protein Data Bank, however, the sequence identity of these Lol proteins to *Xcc* LolA and LolB are also low, 31% for LolA and 21% for LolB.

Arg43 in *E. coli* LolA is an example of a non-conserved residue that has been assigned an important function in LP transfer to LolB (Kaplan et al., 2022). The LolA variant R43L was able to bind LP and displayed 10-fold higher affinity for LolB compared with wild-type LolA. Crystal structures of LP-bound forms of wild-type LolA and R43L LolA showed that the lipid anchor was bound in different conformations in the two structures, and that LP transfer from R43L LolA to wild-type LolB was abolished (Kaplan et al., 2022). The corresponding residue in *Xcc* LolA is a threonine (Thr43), which does not form any interactions with residues predicted to control lid conformation or lipid binding.

Another important difference concerns the LolB hook. The hook in *Xcc* LolB (residues 74–79) has the sequence ^74^APVSRQ^79^ while the *E. coli* LolB hook sequence is ^66^QPLGS^70^. The *Xcc* LolB hook is one residue longer and has only the proline in common with the *E. coli* LolB hook. Replacing Leu68 in the *E. coli* LolB hook by polar or charged amino acids produced variants that were defective to varying degrees in LP localization (Hayashi et al., 2014). Interestingly, in the *Xcc* LolB hook, Arg79 occupies approximately the same space as Leu68 in *E. coli* LolB, which points to a fundamentally different localization mechanism in *Xcc*.

Of the hydrophobic side chains accessible for interaction with a lipid anchor in the cavities of *Xcc* and *E. coli* LolA and LolB, approximately 20% are conserved for LolA and 29% for LolB, however, with few exceptions, the amino-acid replacements retain the hydrophobic character (Supplementary Table S4). For example, in *E. coli* LolA, Phe16 and Phe140 were found to be important for lipid binding, and replacement of these residues by smaller, polar, or charged side chains inhibited or abolished *E. coli* growth (Kaplan et al., 2022). These residues are not conserved in *Xcc* LolA (Leu16 and Met145) but are hydrophobic and would still be able to interact favorably with an acyl chain. Calculation of the accessible hydrophobic surfaces show a significant difference between *Xcc* LolA and LolB with hydrophobic surfaces of 2,200 Å^2^ and 3,100 Å^2^ of the LolA and LolB cavities, respectively (Supplementary Figure S7). The hydrophobic surface of the cavity in *E. coli* LolA is very similar to that in *Xcc* LolA, and while there is no open experimental or theoretical structure for *E. coli* LolB available to use for calculating the accessible hydrophobic surface, we expect a similar result as for *Xcc* LolB.

When modeling a diacyl lipid anchor to *Xcc* LolB, several aromatic residues are expected to make favorable interactions with the lipid, such as Phe42, Trp61, Trp117, Trp127 (Supplementary Table S4). These correspond to Thr33, Trp52, Met107, and Trp117 in *E. coli* LolB. Mutagenesis of *E. coli* LolB Trp52 (W52P) and Trp117 (W117A) produced LolB variants that were defective in lipid receptor activity (Hayashi et al., 2014), which supports the importance of these residues for binding.

By comparing the conformational states of *Xcc* and *E. coli* LolA and LolB, we conclude that the minor conformational changes are required to accommodate a lipid anchor in the cavities, and as described above, it is sufficient to move the helical lids (Supplementary Figure S8). The open lipid-bound state of *E. coli* LolB was not possible to evaluate since only a semi-open state with a bound polyethylene glycol (PEG) molecule is available (PDB 1IWN; Takeda et al., 2003), but we predict that the lid movement in *E. coli* LolB would be very similar to that predicted for *Xcc* LolB.

Thus, while the regions in LolA and LolB that form the AB interface in *Xcc* and *E. coli* coincide and the overall association mode is expected to be similar, the precise structural determinants that provide the basis of lid stabilization, lipid binding, AB association, hook interactions and transfer mechanism are different.

## Discussion

### *Xcc* LolA and LolB forms a stable complex in solution in the absence of lipoprotein

Our results from SEC and ITC analyses show that *Xcc* LolA and a periplasmic variant of LolB form a 1:1 complex in solution in the absence of bound lipoprotein (LP). For *E. coli* LolB, a periplasmic version (mLolB) was able to successfully accept lipoprotein cargo from LolA but caused mislocalization of OM lipoproteins to the inner membrane (Tsukahara et al., 2009). Thus, at least in *E. coli*, the absence of the tether peptide does not appear to influence lipid binding or transfer between the two proteins. In our study, we also used a periplasmic version of *Xcc* LolB, which in analogy with *E. coli* is expected to be functionally competent.

The crystal contains two LolA-LolB heterodimers in the asymmetric unit, but results from the SEC analysis confirmed an AB assembly (LolA-LolB complex). Based on ITC measurements, the association of *Xcc* LolA and LolB association has a *K*_d_ value of about 15 μM, which is in a similar range as that observed for the *E. coli* complex of 31.5 μM (Kaplan et al., 2022). Besides the proteins being of different bacterial origin and physicochemical properties, the precise value of the dissociation constant will depend on the *in vitro* conditions for the measurements, and hence the value can only be regarded to an estimate.

The crystal structure allowed us to identify interactions that stabilize the LP-free *Xcc* LolA-LolB complex that do not coincide with the residues predicted to bind the lipid anchor. These interactions map to the C-terminal strand β12A and β5A/β6A loop in LolA; and residues in β1B, β2B, β10B and β11B in LolB (Figure 4; Supplementary Table S3). The importance of the C-terminal strand β12A has been proposed also for *E. coli* LolA based on mutagenesis studies where β12A was found to be essential for the release and transfer of LPs (Okuda et al., 2008). Furthermore, photo-crosslinking experiments were performed to identify the interacting surfaces of *E. coli* LolA and LolB (Okuda and Tokuda, 2009), which showed roughly the same regions as those observed in the *Xcc* complex.

### Docking of LP-bound LolA with LolB to simulate the pre-transfer state

Docking of a diacyl lipid to LolA requires opening of the helical lid and the β8A/β9A loop. Upon docking, the LolB hook (residues 74–79) needs to insert between one of the acyl chains (probably R2) and the inner β-sheet 2 in LolA. Based on our modeling, this should be feasible in the predicted LP-bound “open” state of LolA where the helical lid is open and the β8A/β9A loop displaced by 3–4 Å compared to the closed unbound LolA state.

In addition to the AB-specific interactions that stabilize the LolA-LolB complex (Figure 4; Supplementary Table S3), the conformation of the LolB hook is expected to affect the precise association of LolA and LolB. This becomes evident when comparing the two experimental LolA-LolB complexes in the crystal where the AB-specific interactions are preserved but the hook has different conformations (Supplementary Figure S5A). The different conformation of the hook introduces small but distinct changes in the relative positions of LolA and LolB, which results in a somewhat tighter association for heterodimer 1 (chains A and B) compared to heterodimer 2 (chains C and D). This is supported by *PDBePISA*, which predicted a less stable assembly of heterodimer 2. Thus, the LP-free complex is stabilized by AB-specific interactions, but the stability is modulated by the hook conformation.

In our theoretical model of LP-bound LolA and LolB, the AB-specific interactions (Supplementary Table S3) would be possible to maintain, but the overall stability of the complex is expected to be weakened due to the LolB hook chiseling in between the lipid and inner β-sheet 2 in LolA (Supplementary Figure S5B). Thus, in this simulated docking state of LP-bound LolA and LolB, the AB-specific interactions would transiently stabilize the complex to promote association to enable docking of the LolB hook.

### Transfer of LP from LolA to LolB

Although the β-barrel folds of LolA and LolB are similar, their helical lids are very different both structurally and how they shield the hydrophobic cavities. The lid in LolA is further away from the AB interface and therefore, conformational changes of the LolB lid would impact the integrity of the AB heterodimer to a larger extent. We predict that at the docking event, the process of the LolB hook chiseling in between the lipid and inner LolA β-sheet would initiate a series of structural changes starting with residues in the LolB hook and transmitting deeper into LolB via the β-strands immediately before and after the hook (β3B and β4B) (Supplementary Figure S9).

Based on our predicted model of the post-transfer state of LolB where the LolB lid is open to accommodate the diacyl lipid, we predict that it will become increasingly difficult to maintain the AB association mode as the hook-initiated conformational changes take place that leads to progressive opening of the LolB lid. Simultaneous weakening of the LolA-LolB interactions and formation of hydrophobic interactions between the hook and the diacyl lipid would allow the hook to “scoop out” the lipid from LolA while the peptide region of the diacyl anchor being secured by polar interactions provided by side chains that cluster at the LolB side of the rim of the annulus observed for the unbound AB complex. Thus, LP-bound LolA would remain associated with LolB for only as long as is required to initiate LP transfer and the concomitant release of LolA.

### Possible implications of an LP-free LolA and LolB complex

The observation of a stable, LP-free *Xcc* LolA-LolB complex is interesting since we expected that in the absence of lipid, the interactions between LolA and LolB would be weak, or even non-existing. Rather, *Xcc* LolA and LolB evolved PPIs that enable complex formation in the absence of bound cargo. This implies that for a successful initial docking event to take place between LP-bound LolA and LolB, PPIs that are independent from lipid-binding interactions are probably required for association and productive lipid transfer. Stable PPIs between LolA and LolB would increase the probability of successful docking and grant a sufficiently long-lived association state to ensure productive transfer.

The current paradigm of lipoprotein transfer does not address the fate of LolA, or LolB, after delivering the LP cargo. The formation of stable LP-free LolA-LolB complexes raises the question of whether LolA, after delivering its cargo to LolB, could be captured by available LP-free LolB molecules attached to the OM. We expect LP-bound LolA to have a thermodynamic advantage over LP-free LolA in the encounter with such species, and that their presence would not obstruct the continuous productive transfer of LP from LolA to LolB. Further investigations are required to shed further light on the fate of LolA after LP delivery, and if there is any biological relevance of LP-free LolA-LolB complexes beyond safeguarding unidirectional LP transfer from LolA to LolB.

## Data availability statement

The datasets presented in this study can be found in online repositories. The names of the repository/repositories and accession number(s) can be found at: https://www.rcsb.org/, 8ORN.

## Author contributions

VF and CD designed and performed experiments and analyzed the data. CD conceived the research. All authors contributed to the article and approved the submitted version.

## Funding

This work was supported by the Swedish Research Council Formas (Grant number 2017–00983) and Oscar and Lili Lamm Memorial Foundation (Grant number DO2017-0020) to CD.

## Conflict of interest

The authors declare that the research was conducted in the absence of any commercial or financial relationships that could be construed as a potential conflict of interest.

## Publisher’s note

All claims expressed in this article are solely those of the authors and do not necessarily represent those of their affiliated organizations, or those of the publisher, the editors and the reviewers. Any product that may be evaluated in this article, or claim that may be made by its manufacturer, is not guaranteed or endorsed by the publisher.

## Footnotes

^1^https://www.uniprot.org
