# Supplementary material for: LolA and LolB from the plant-pathogen Xanthomonas campestris forms a stable heterodimeric complex in the absence of lipoprotein
Source: Front Microbiol. 2023 Jul 12;14:1216799. doi: 10.3389/fmicb.2023.1216799 (PMC10368991; doi:10.3389/fmicb.2023.1216799)
Supplement: Supplementary file 1 [file Data_Sheet_1.PDF]

Supplementary Material

LolA and LolB from the plant-pathogen *Xanthomonas campestris* forms a stable heterodimeric complex in the absence of lipoprotein

Valentina Furlanetto, Christina Divne*

*** Correspondence:**

Corresponding Author: divne@kth.se

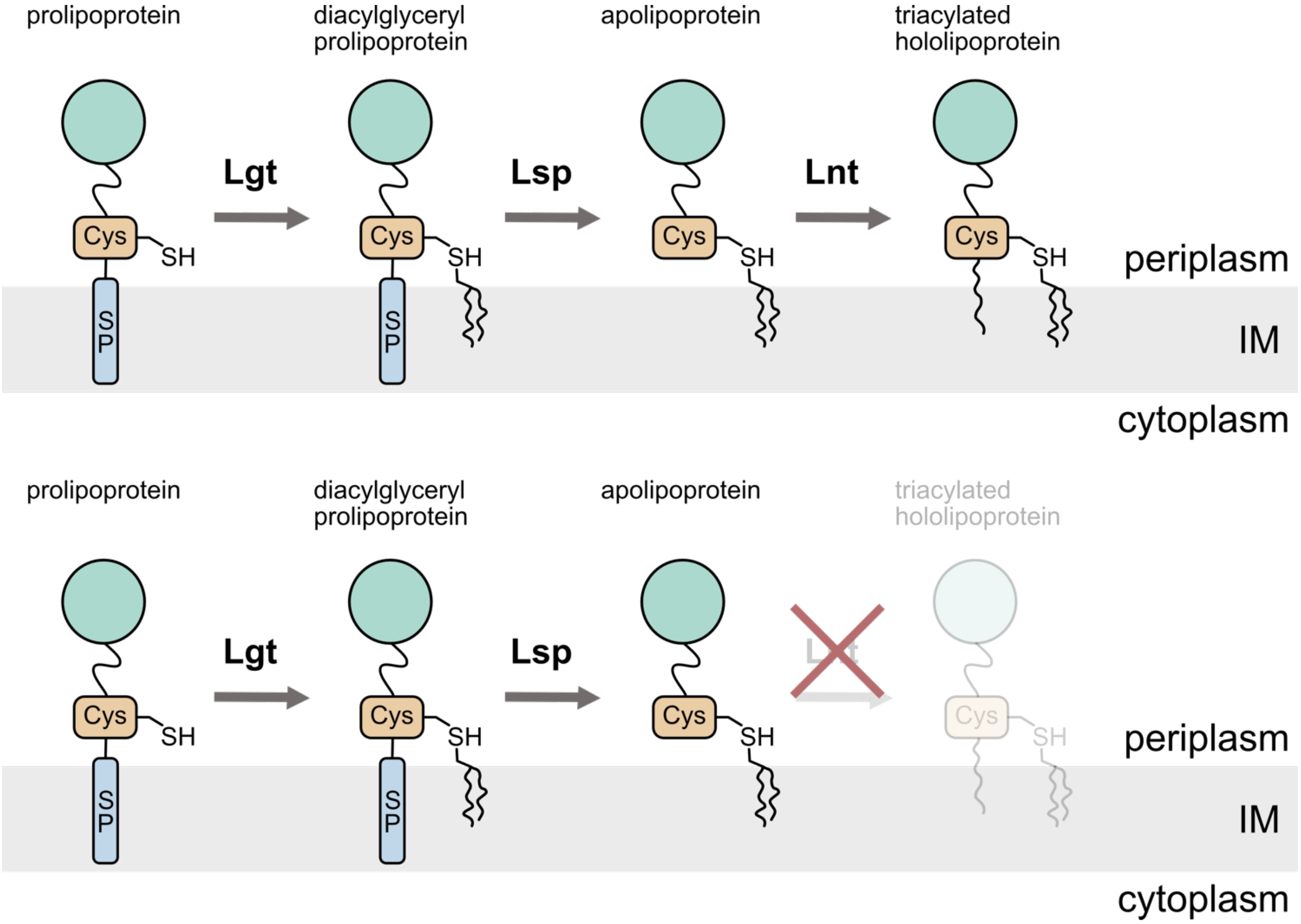

Supplementary Figure 1. Synthesis of the lipoprotein acyl anchor. Upper pathway represents the synthesis of a triacylated lipoprotein where Lgt attaches a diacylglycerol to the cysteine of the prolipoprotein *via* a thioester bond to produce a diacylglyceryl prolipoprotein, Lsp cleaves off the N-terminal lipoprotein signal sequence to produce the apolipoprotein, and finally, Lnt adds the third acyl chain *via* an amide bond to the cysteine to generate the mature triacylated hololipoprotein. The lower pathway represents the assumed pathway for bacteria that lack Lnt, thus abolishing the addition of the third acyl chain.

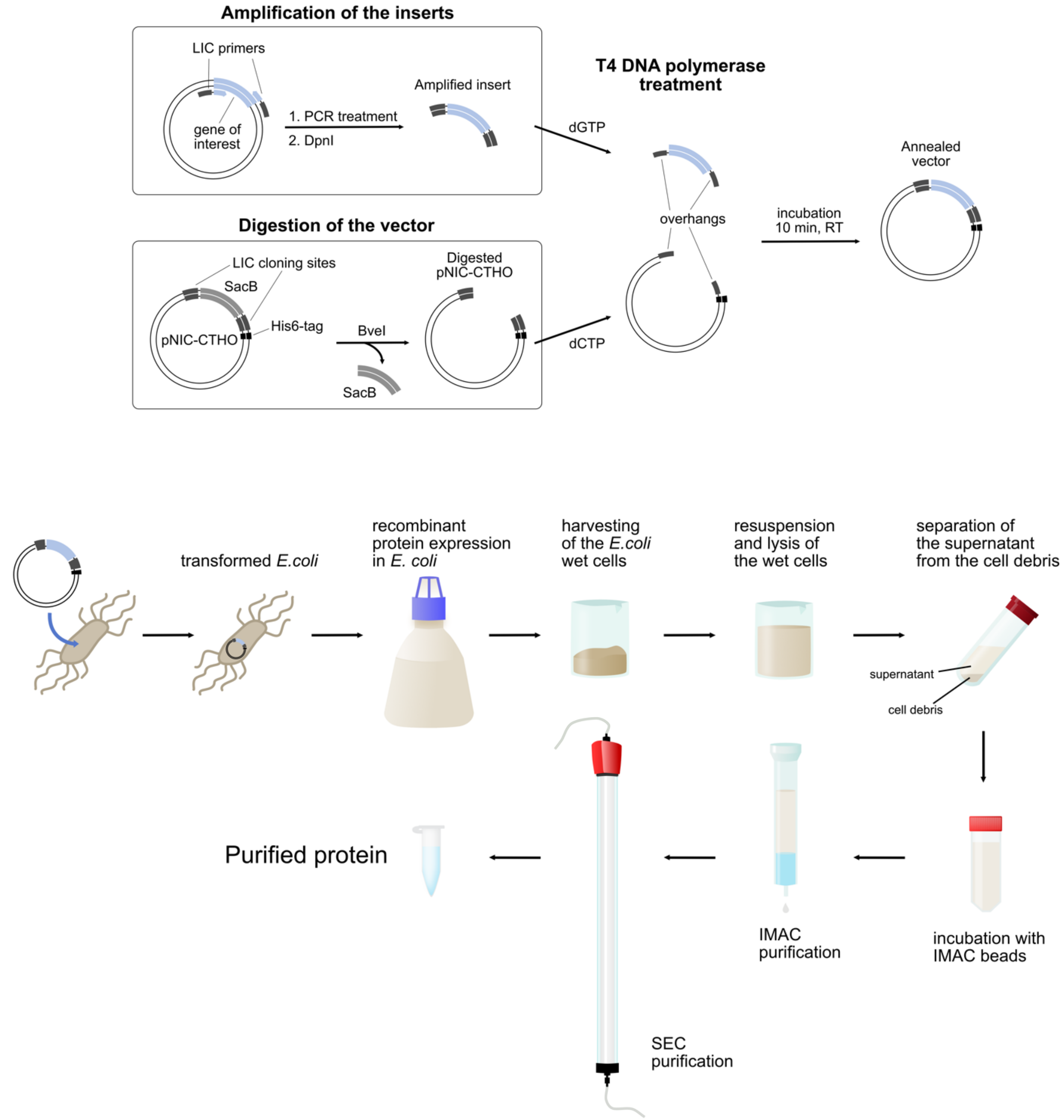

Supplementary Figure 2. Workflow for gene cloning and heterologous protein production in *E. coli*. Ligation-independent cloning protocol including amplification of the gene insert, synthesis of the nucleotide overhangs and annealing with the vector. The recombinant vector was transformed into *E. coli* cells, followed by bacterial growth and expression of the gene insert. Harvested cells were lysed and the overexpressed gene product (LolA or LolB) was purified by Ni^{2+}-IMAC and SEC. See text for further details.

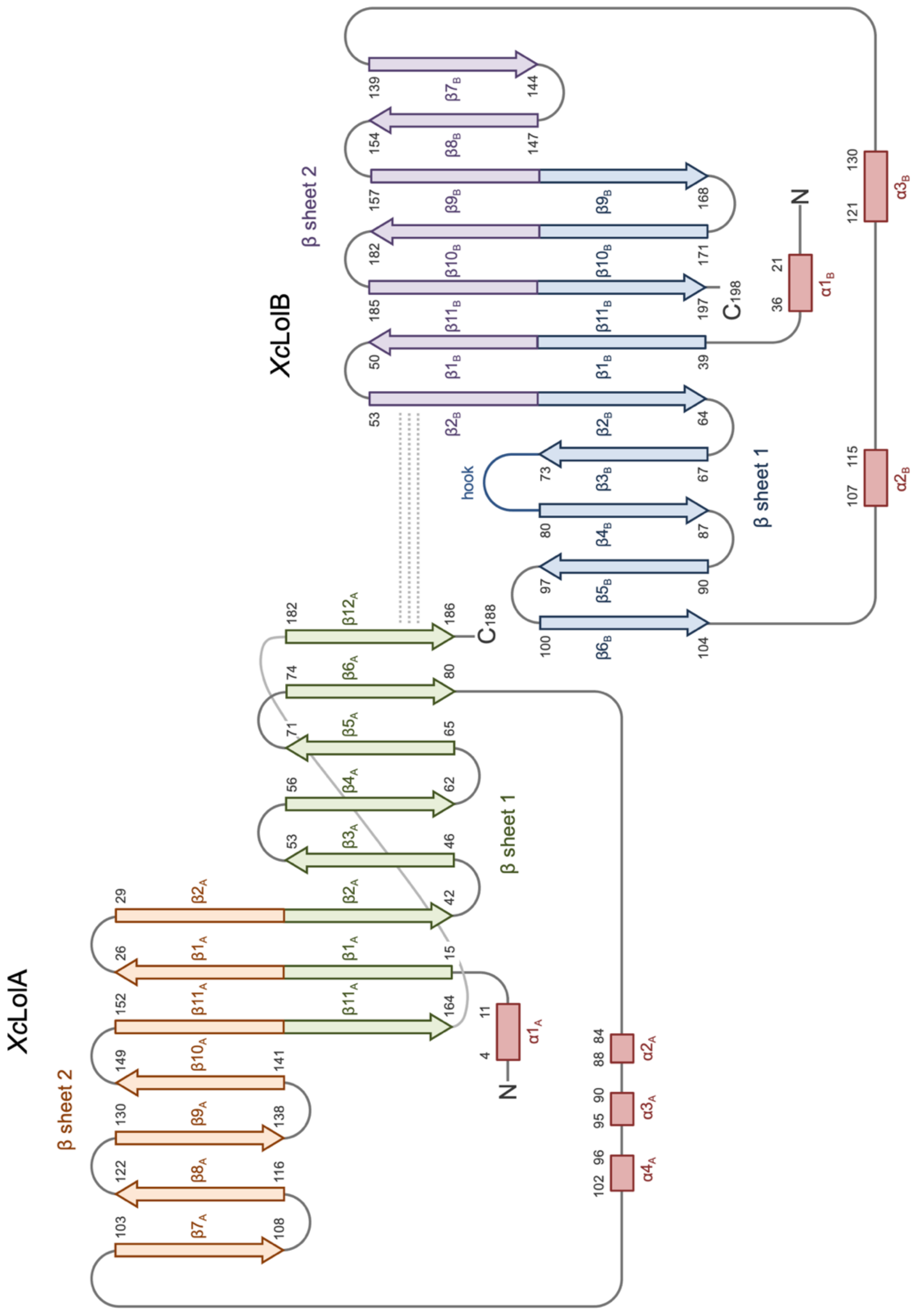

Supplementary Figure 3. Topology diagrams of *Xcc* LolA and LolB. *Xcc* LolA and LolB each contains two β-sheets (sheets 1 and 2). Secondary structure elements in *Xcc* LolA and *Xcc* LolB have subscripts A and B, respectively. The topology diagram of LolB has been rotated to show how sheet 1 in LolA interacts with sheet 2 in LolB *via* hydrogen bonds formed between strands β12A and β2B that run parallel to each other. The lid that controls access to the hydrophobic cavity in LolA includes helices α2A-α4A, and the corresponding lid in LolB includes helices α2B-α3B.

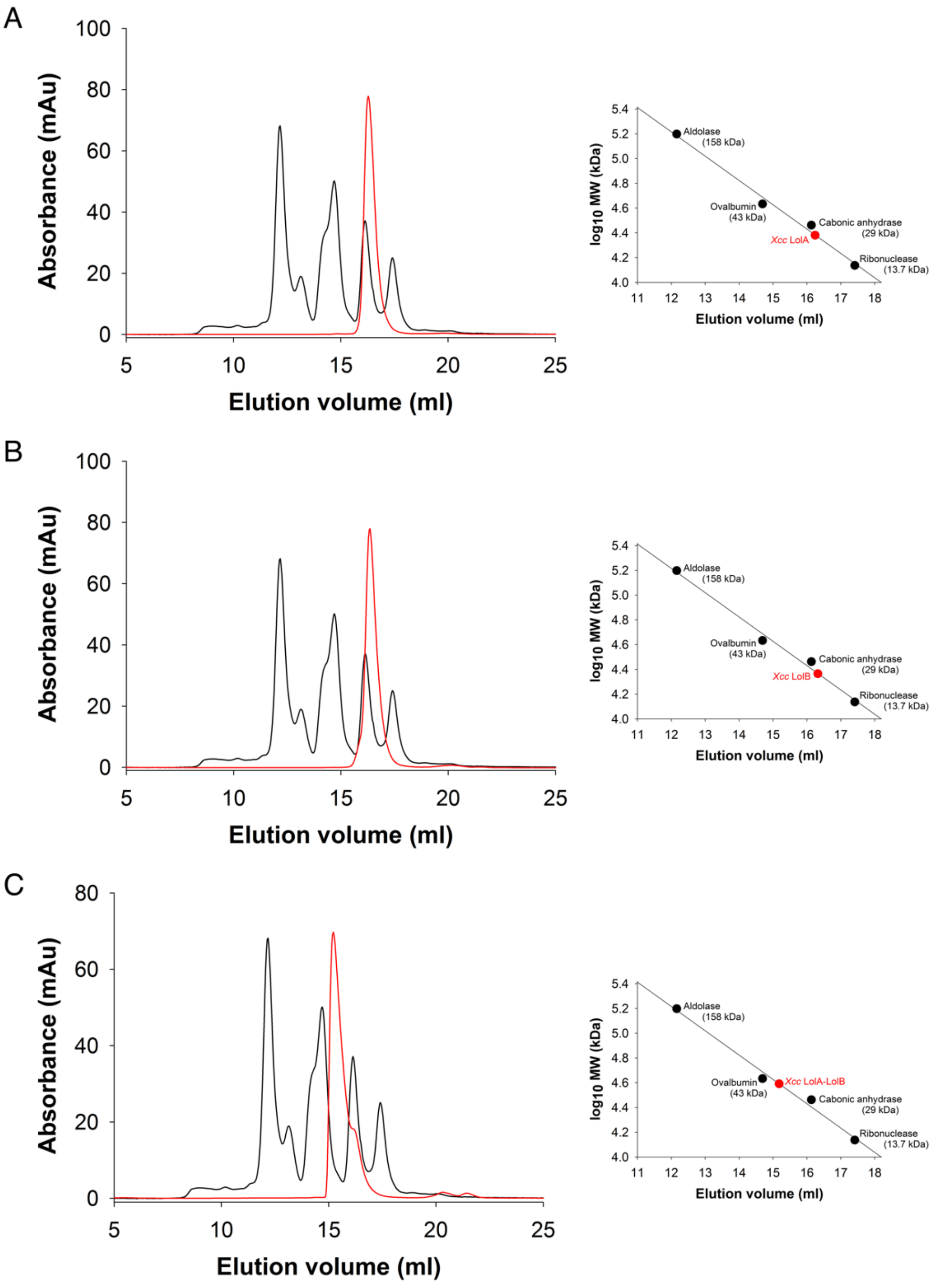

Supplementary Figure 4. Size-exclusion chromatography analysis. SEC profiles for (**A**) *Xcc* LolA, (**B**) *Xcc* LolB, and (**C**) *Xcc* LolA-LolB complex.

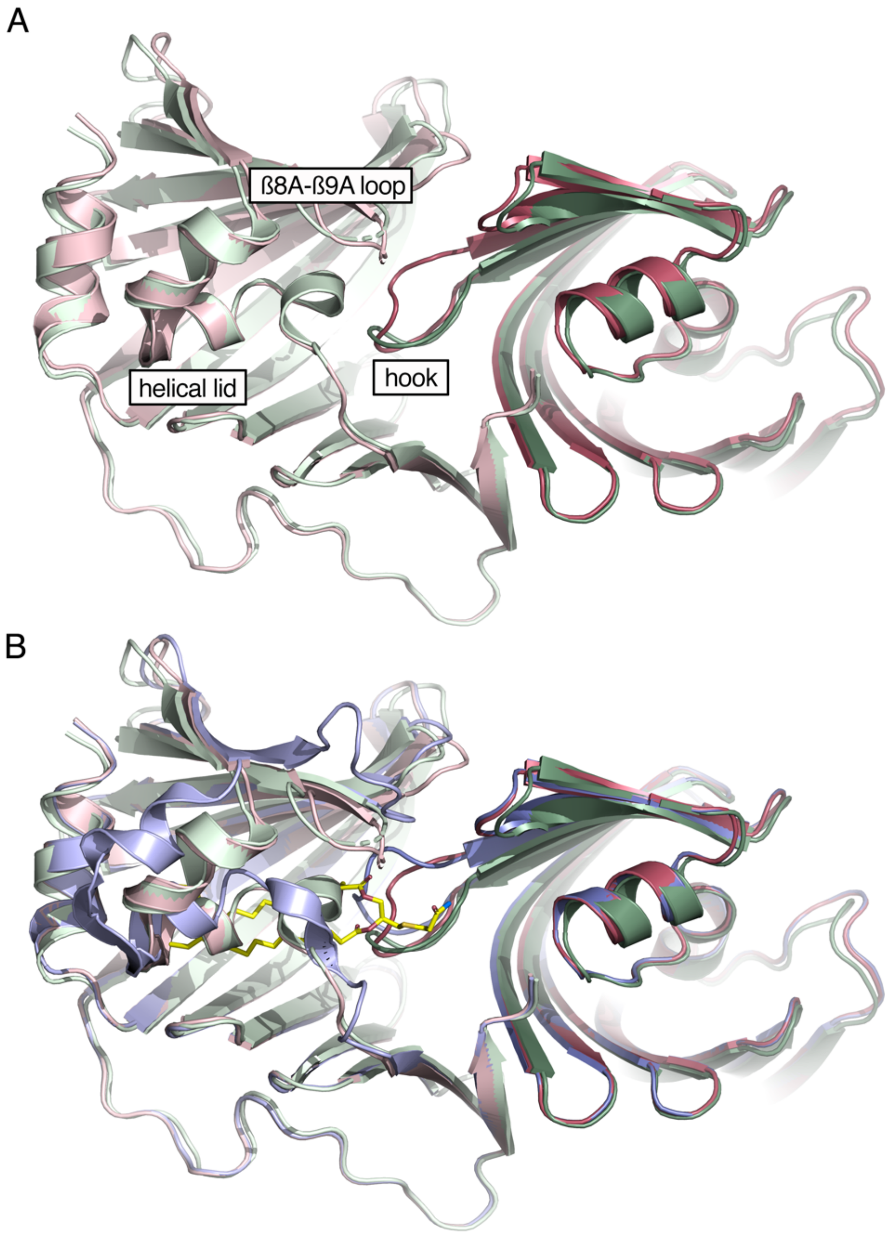

Supplementary Figure 5. Comparison of LolA-LolB heterodimers. (**A**) Superposition of the two lipid-free LolA-LolB heterodimers of the asymmetric unit in the crystal structure: heterodimer 1 with LolA (chain A) in light pink and LolB (chain B) in dark pink; and heterodimer 2 with LolA (chain C) in light green and LolB (chain D) in dark green. The lolB hook is positioned at the center of the picture and assumes different conformation in the two heterodimers, which produces concomitant shifts of β-strands and connecting loops in β-sheet 2 of LolA. (**B**) Same as in (A) but adding a theoretical model of the LolA-LolB complex with a lipid anchor bound to LolA (LolA in light blue and LolB in dark blue). The figure was prepared using PyMOL 2.4.2 (The PyMOL Molecular Graphics System, Version 2.0 Schrödinger, LLC).

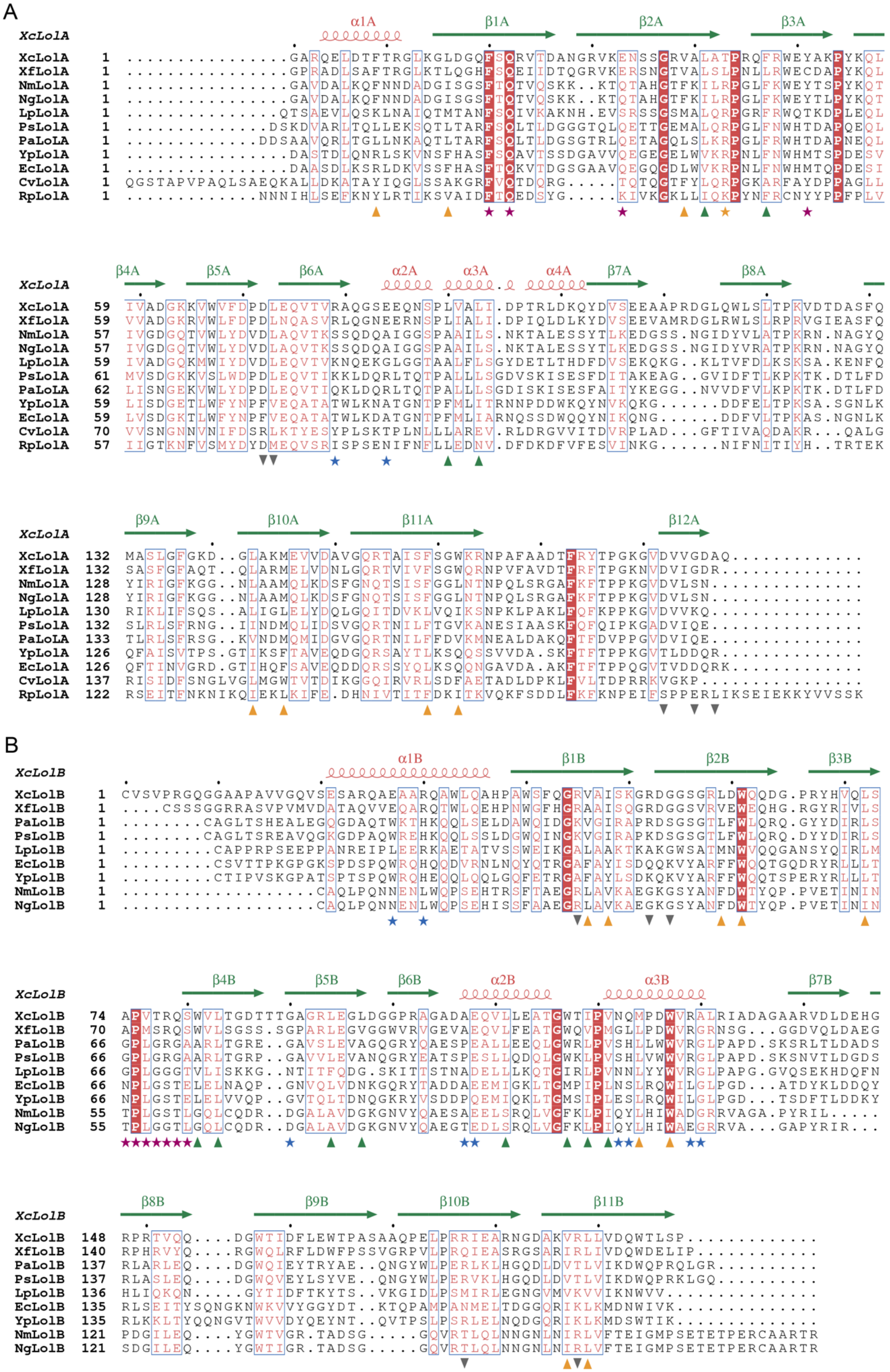

A
α1A β1A β2A β3A
XcLolA
XcLolA 1 ................GARQELDTFTRGLKGLDGQFSQRVTDANGRVKENSSGRVALATPRQFRWEYAKPYKQL
XfLolA 1 ................GPRADLSAFTRGLKTLQGHFSQEIIDTQGRVKERSNGTVALSLPNLLRWECDAPYKQL
NmLolA 1 ................GAVDALKQFNNDADGISGSFTQTVQSKK..KTQTAHGTFKILRPGLFKWEYTSPYKQT
NgLolA 1 ................GAVDALKQFNNDADGISGSFTQTVQSKK..KTQTAHGTFKILRPGLFKWEYTLPYKQT
LpLolA 1 ...............QTSAEVLQSKLNAIQTMTANFSQIVKAKNH.EVSRSSGSMALQRPGRFRWQTKDPLEQL
PsLolA 1 ..............DSKDVARLTQLLEKSQTLTARFSQLTLDGGGTQLQETTGEMALQRPGLFNWHTDAPQEQL
PaLoLA 1 .............DDSAAVQRLTGLLNKAQTLTARFSQLTLDGSGTRLQETAGQLSLKRPGLFRWHTDAPNEQL
YpLolA 1 ................DASTDLQNRLSKVNSFHASFSQAVTSSDGAVVQEGEGELWVKRPNLFNWHMTSPDESV
EcLolA 1 ................DAASDLKSRLDKVSSFHASFTQKVTDGSGAAVQEGQGDLWVKRPNLFNWHMTQPDESI
CvLolA 1 QGSTAPVPAQLSAEQKALLDKATAYIQGLSSAKGRFVQTDQRG.....TQTQGTFYLQRPGKARFAYDPPAGLL
RpLolA 1 .............NNNIHLSEFKNYLRTIKSVAIDFTQEDSYG.....KIVKGKLLIQKPYNFRCNYYPPFPLV
β4A β5A β6A α2A α3A α4A β7A β8A
XcLolA
XcLolA 59 IVADGKKVWVFDPDLEQVTVRAQGSEEQNSPLVALI.DPTRLDKQYDVSEEAAPRDGLQWLSLTPKVDTDASFQ
XfLolA 59 VVADGKRVWLFDPDLNQASVRLQGNEERNSPLIALI.DPIQLDLKYDVSEEVAMRDGLRWLSLRPRVGIEASFQ
NmLolA 57 IVGDGQTVWLYDVDLAQVTKSSQDQAIGGSPAAILS.NKTALESSYTLKEDGSS.NGIDYVLATPKRN.NAGYQ
NgLolA 57 IVGDGQTVWLYDVDLAQVTKSSQDQAIGGSPAAILS.NKTALESSYTLKEDGSS.NGIDYVRATPKRN.NAGYQ
LpLolA 59 IVADGQKMWIYDVDLEQVTVKNQEKGLGGTAALFLSGYDETLTHDFDVSEKQKG..KLTVFDLKSKSA.KENFQ
PsLolA 61 MVSDGKKVSLWDPDLEQVTIKKLDQRLTQTPALLLSGDVSKISESFDITAKEAG..GVIDFTLKPKTK.DTLFD
PaLoLA 62 LISNGEKVWLYDPDLEQVTIQKLDQRLTQTPALLLSGDISKISESFAITYKEGG..NVVDFVLKPKTK.DTLFD
YpLolA 59 LISDGETLWFYNPFVEQATATWLKNATGNTPFMLITRNNPDDWKQYNVKQKG......DDFELTPKSA.SGNLK
EcLolA 59 LVSDGKTLWFYNPFVEQATATWLKDATGNTPFMLIARNQSSDWQQYNIKQNG......DDFVLTPKAS.NGNLK
CvLolA 70 VVSNGNNVNIFDSRLKTYESYPLSKTPLNLLLAREV.RLDRGVVITDVRPLAD...GFTIVAQDAKR...QALG
RpLolA 57 IIGTKNFVSMYDYDMEQVSRISPSENIFNFLLEDNV.DFDKDFVFESVINKG......NIFNITIYH..TRTEK
β9A β10A β11A β12A
XcLolA
XcLolA 132 MASLGFGKD..GLAKMEVVDAVGQRTAISFSGWKRNPAFAADTFRYTPGKGVDVVGDAQ.............
XfLolA 132 SASFGFAQT..QLARMELVDNLGQRTVIVFSGWQRNPVFAVDTFRFTPGKNVDVIGDR..............
NmLolA 128 YIRIGFKGG..NLAAMQLKDSFGNQTSISFGGLNTNPQLSRGAFKFTPPKGVDVLSN...............
NgLolA 128 YIRIGFKGG..NLAAMQLKDSFGNQTSISFGGLNTNPQLSRGAFKFTPPKGVDVLSN...............
LpLolA 130 RIKLIFSQS..ALIGLELYDQLGQITDVKLVQIKSNPKLPAKLFQFKPPKGVDVVKQ...............
PsLolA 132 SLRLSFRNG..IINDMQLIDSVGQRTNILFTGVKANESIAASKFQFQIPKGADVIQE...............
PaLoLA 133 TLRLSFRSG..KVNDMQMIDGVGQRTNILFFDVKMNEALDAKQFTFDVPPGVDVIQE...............
YpLolA 126 QFAISVTPS.GTIKSFTAVEQDGQRSAYTLKSQQSSVVDA.SKFTFTPPKGVTLDDQR..............
EcLolA 126 QFTINVGRD.GTIHQFSAVEQDDQRSSYQLKSQQNGAVDA.AKFTFTPPQGVTVDDQRK.............
CvLolA 137 RISIDFSNGLVGLMGWTVTDIKGGQIRVRLSDFAETADLDPKLFVLTDPRRKVGKP................
RpLolA 122 RSEITFNKNIKQIEKLKIFE.DHNIVTITFDKITKVQKFSDDLFKFKNPEIFSPPERLIKSEIEKKYVVSSK
B
α1B β1B β2B β3B
XcLolB
XcLolB 1 CVSVPRGQGGAAPAVVGQVSESARQAEAARQAWLQAHPAWSFQGRVAISKGRDGGSGRLDWQQDG.PRYHVQLS
XfLolB 1 ....CSSSGGRRASVPVMVDATAQVVEQARQTWLQEHPNWGFHGRAAISQGRDGGSVRVEWEQHG.RGYRIVLS
PaLolB 1 .........CAGLTSHEALEGQGDAQTWKTHKQQLSELDAWQIDGKVGIRAPRDSGSGTLFWLQRQ.GYYDIRLS
PsLolB 1 .........CAGLTSREAVQGKGDPAQWREHKQQLSSLDGWQINGKVGIRAPKDSGSGTLFWLQRQ.DYYDIRLS
LpLolB 1 ..........CAPPRPSEEPPANREIPLEERKAETATVSSWEIKGALAAKTKAKGWSATMNWVQQGANSYQIRLM
EcLolB 1 ..........CSVTTPKGPGKSPDSPQWRQHQQDVRNLNQYQTRGAFAYISDQQKVYARFFWQQTGQDRYRLLLT
YpLolB 1 ..........CTIPVSKGPATSPTSPQWRQHEQQLQQLGQFETRGAFAYLSDKQKVYARFFWQQTSPERYRLLLT
NmLolB 1 ..................CAQLPQNNENLWQPSEHTRSFTAEGRLAVKAEGKGSYANFDWTYQP.PVETININ
NgLolB 1 ..................CAQLPQNNENLWQPSEHISSFAAEGRLAVKAEGKGSYANFDWTYQP.PVETININ
β4B β5B β6B α2B α3B β7B
XcLolB
XcLolB 74 APVTRQSWVLTGDTTTGAGRLEGLDGGPRAGADAEQVLLEATGWTIPVNQMPDWVRALRIADAGAARVDLDEHG
XfLolB 70 APMSRQSWVLSGSS.SGPARLEGVGGWVRVGEVAEQVLFEATGWQVPMGLLPDWVRGRNSG...GGDVQLDAEG
PaLolB 66 GPLGRGAARLTGRE..GAVSLEVAGQGRYQAESPEALLEEQLGWRLPVSHLLWWVRGLPAPD.SKSRLTLDADS
PsLolB 66 GPLGRGAARLTGRP..GAVVLEVANQGRYEATSPESLLQDQLGWKLPVSHLVWWVRGLPAPD.SKSNVTLDGDS
LpLolB 66 GPLGGGTVLISKKG..NTITFQDG.SKITTSTNADELLLKQTGIRLPVNNLYYWVRGLPAPG.GVQSEKHDQFN
EcLolB 66 NPLGSTELELNAQP..GNVQLVDNKGQRYTADDAEEMIGKLTGMPIPLNSLRQWILGLPGD...ATDYKLDDQY
YpLolB 66 NPLGSTELELVVQP..GVTQLTDNQGKRYVSDDPQEMIQKLTGMSIPLESLRQWILGLPGD...TSDFTLDDKY
NmLolB 55 TPLGSTLGQLCQDR..DGALAVDGKGNVYQAESAEELSRQLVGFKLPIQYLHIWADGRRVAGA.PYRIL.....
NgLolB 55 TPLGGTLGQLCQDR..DGALAVDGKGNVYQAEGTEDLSRQLVGFKLPIQYLHIWAEGRRVA...GAPYRIR...
β8B β9B β10B β11B
XcLolB
XcLolB 148 RPRTVQQ....DGWTIDFLEWTPASAAQPELPRRIEARNGDAKVRLLVDQWTLSP.............
XfLolB 140 RPHRVYQ....RGWQLRFLDWFPSSVGRPVLPRQIEASRGSARIRLIVDQWDELIP............
PaLolB 137 RLARLEQ....DGWQIEYTRYAE..QNGYWLPERLKLHGQDLDVTLVIKDWQPRQLGR..........
PsLolB 137 RLASLEQ....DGWQVEYLSYVE..QNGYWLPERVKLHGQDLDVTLVIKDWQPRKLGQ..........
LpLolB 136 HLIQKQN.....GYTIDFTKYTS..VKGIDLPSMIRLEGNGVMVKVVIKNWVV...............
EcLolB 135 RLSEITYSQNGKNWKVVYGGYDT..KTQPAMPANMELTDGGQRIKLKMDNWIVK..............
YpLolB 135 RLKKLTYQQNGVTWVVDYQEYNT..QVTPSLPSRLELNQDGQRIKLKMDSWTIK..............
NmLolB 121 PDGILEQ....YGWTVGR.TADSG.....GQVRTLQLNNGNLNIRLVFTEIGMPSETETPERCAARTR
NgLolB 121 SDGILEQ....YGWTIGR.TADSG.....GQVRTLQLNNGNLNIRLVFTEIGMPSETETPERCAARTR

Supplementary Figure 6. Multiple-sequence alignment of LolA and LolB protein sequences. Alignment of a selection of LolA and LolB sequences from pathogenic Gram-negative bacteria. The naming convention of secondary-structure elements is the same as in Supplementary Figure 3. UniProt accession codes for the aligned sequences are given in Supplementary Table 5. (**A**) *Xc*LolA, *Xanthomonas campestris* pv. *campestris*; *Xf*LolA, *Xylella fastidiosa*; *Nm*LolA, *Neisseria meningitidis*; *Ng*LolA, *Neisseria gonorrhoeae*; *Lp*LoLA, *Legionella pneumophila*; *Ps*LolA, *Pseudomonas syringae*; *Pa*LoLA, *Pseudomonas aeruginosa*; *Yp*LolA, *Yersinia pestis*; *Ec*LolA, *Escherichia coli*; *Cv*LolA, *Caulobacter vibrioides*; *Rp*LolA, *Rickettsia prowazekii*. Highlighted functional residues in *Xc*LolA: orange up-triangles, hydrophobic side chains predicted to contact the R1 acyl chain; hydrophobic side chains predicted to contact the R2 acyl chain, green up-triangles; gray down-triangles; residues in LolA that participate in stabilization of the LolA-LolB complex; purple stars, LolB hook; blue stars, residues that control the lid in LolA. (**B**) *Xc*LolB, *X. campestris* pv. *campestris*; *Xf*LolB, *X. fastidiosa*; *Pa*LolB, *P. aeruginosa*; *Ps*LolB, *P. syringae*; *Lp*LolB, *L. pneumophila*; *Ec*LolB, *E. coli*; *Yp*LolB, *Y. pestis*; *Nm*LolB, *N. meningitidis*; *Ng*LolB, *N. gonorrhoeae*. The predicted sequences for the periplasmic signal peptide have been removed from all LolA sequences, and the predicted lipoprotein signal peptides removed from all LolB sequences. *C. vibrioides* and *R. prowazekii* do not have LolB homologs. Highlighted functional residues in *Xc*LolB: orange up-triangles, hydrophobic side chains predicted to contact the R1 acyl chain; green up-triangles, hydrophobic side chains predicted to contact the R2 acyl chain; gray down-triangles; residues in LolB that participate in stabilization of the LolA-LolB complex; purple stars, hook; blue stars, residues that control the lid in LolB.

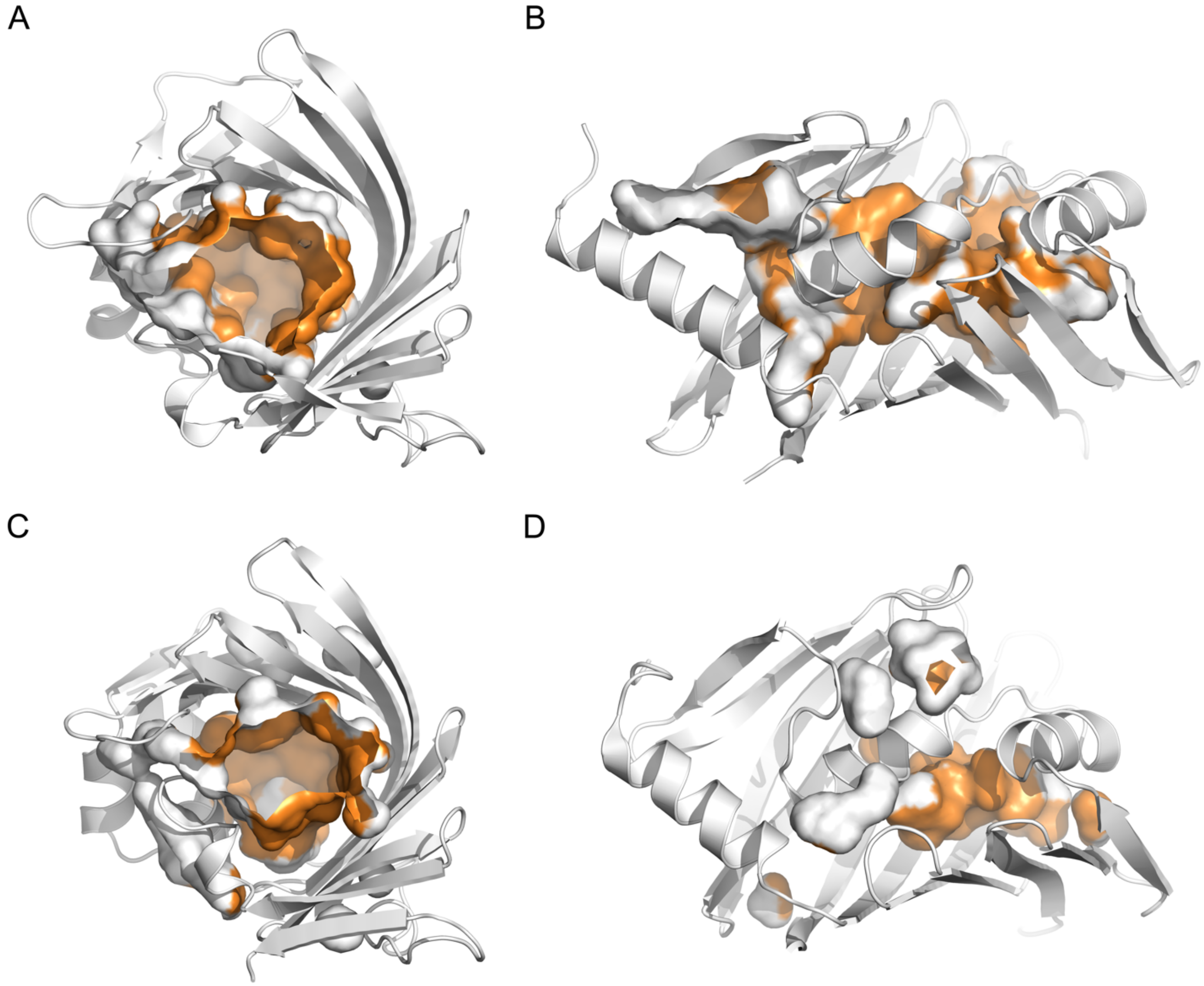

Supplementary Figure 7. Visualization of the accessible hydrophobic surfaces within the cavities of *Xcc* and *E. coli* LolA and LolB cavities. (**A**) *Xcc* LolA in the open state looking into the cavity from a viewpoint at the opening. The helical lid is positioned at the left in the picture. This state corresponds to *Xcc* LolA with a modeled diacyl anchor. (**B**) *Xcc* LolB in the open state with a view perpendicular to the cavity to show its length. This state corresponds to *Xcc* LolB with a modeled diacyl anchor. (**C**) *E. coli* LolA in the open state with a bound triacyl anchor (PDB 7Z6W; Kaplan et al., 2022). *E. coli* LolB in the partly open state with a bound PEG molecule (PDB 1IWN; Takeda et al., 2003). The opening of *E. coli* LolB is closed but is partly open at the far end. (C) and (D) have the same view as (A) and (B), respectively. The cavities were calculated using PyMOL 2.4.2 (The PyMOL Molecular Graphics System, Version 2.0 Schrödinger, LLC). The lipids were removed prior to cavity calculation. The hydrophobic side chains lining the cavities are colored in orange.

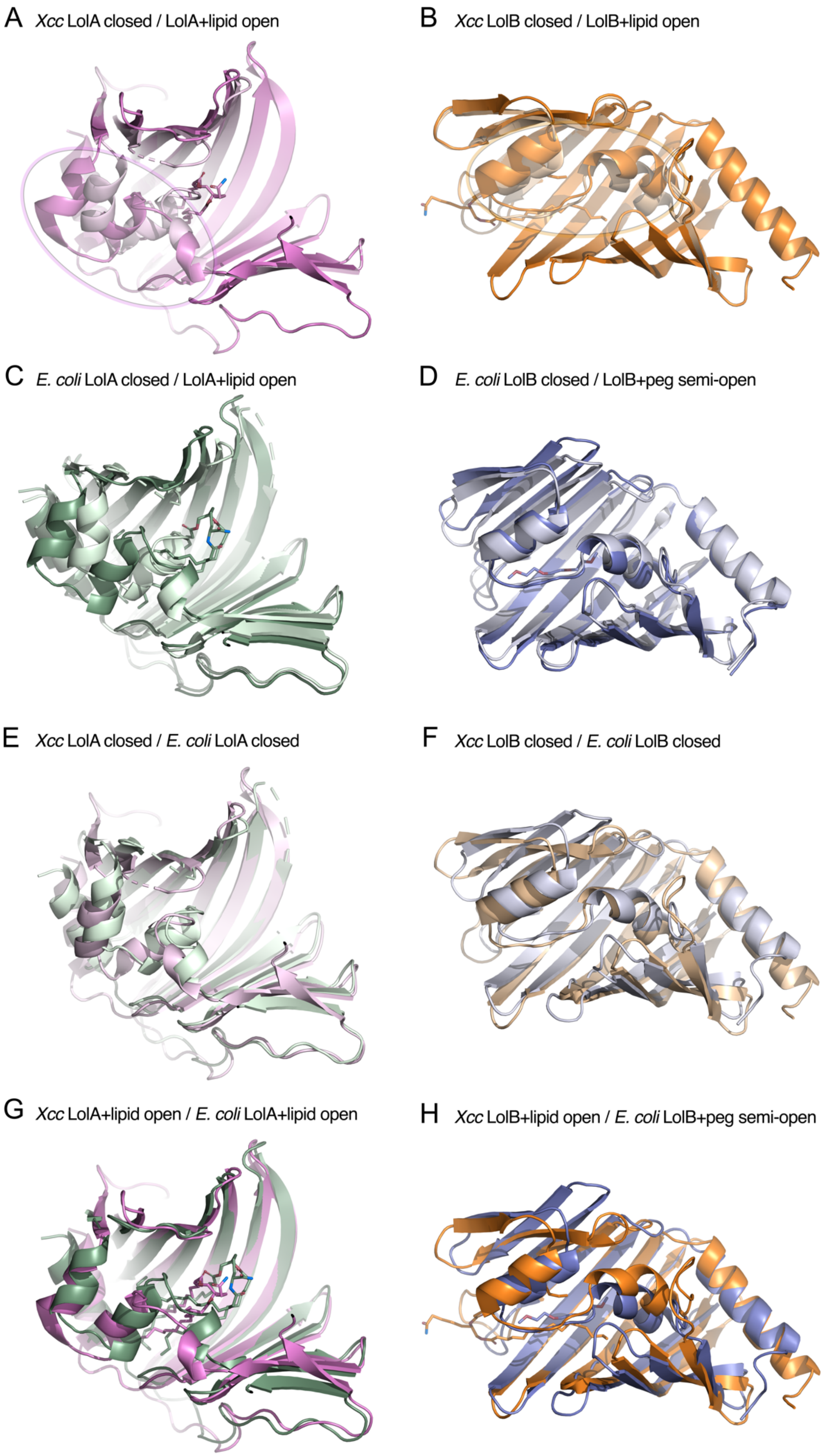
A Xcc LolA closed / LolA+lipid open
B Xcc LolB closed / LolB+lipid open
C E. coli LolA closed / LolA+lipid open
D E. coli LolB closed / LolB+peg semi-open
E Xcc LolA closed / E. coli LolA closed
F Xcc LolB closed / E. coli LolB closed
G Xcc LolA+lipid open / E. coli LolA+lipid open
H Xcc LolB+lipid open / E. coli LolB+peg semi-open

Supplementary Figure 8. Comparison of the proposed closed and lipid-bound states of LolA and LolB from *Xcc* and *E. coli*. Pairwise superpositioning to show the movement of secondary structure elements in response to lipid binding. (**A**) *Xcc* LolA closed state as present in the experimental lipid-free LolA-LolB complex (light pink) and the open state with a modeled diacyl anchor (dark pink). The helical lid is highlighted. (**B**) *Xcc* LolB closed state as present in the experimental lipid-free LolA-LolB complex (light orange) and the open state with a modeled diacyl anchor (dark orange). The helical lid is highlighted. (**C**) *E. coli* LolA in the closed state (light green; PDB 1UA8, Takeda et al., 2003) and in the open state with a bound triacyl anchor (dark green; PDB 7Z6W, Kaplan et al., 2022). (**D**) *E. coli* LolB in the closed state (light blue; PDB 1IWM; Takeda et al., 2003) and in the partly open state with a bound PEG molecule (dark blue; PDB 1IWN, Takeda et al., 2003). (**E**) *Xcc* LolA (light pink) and *E. coli* LolA (light green) in the closed states. (**F**) *Xcc* LolB (light orange) and *E. coli* LolB (light blue) in the closed states. (**G**) *Xcc* LolA (dark pink) and *E. coli* LolA (dark green) in the open states. (**H**) *Xcc* LolB in the open state (dark orange) and *E. coli* LolB in the partly open state (dark blue). The figure was prepared using PyMOL 2.4.2 (The PyMOL Molecular Graphics System, Version 2.0 Schrödinger, LLC).

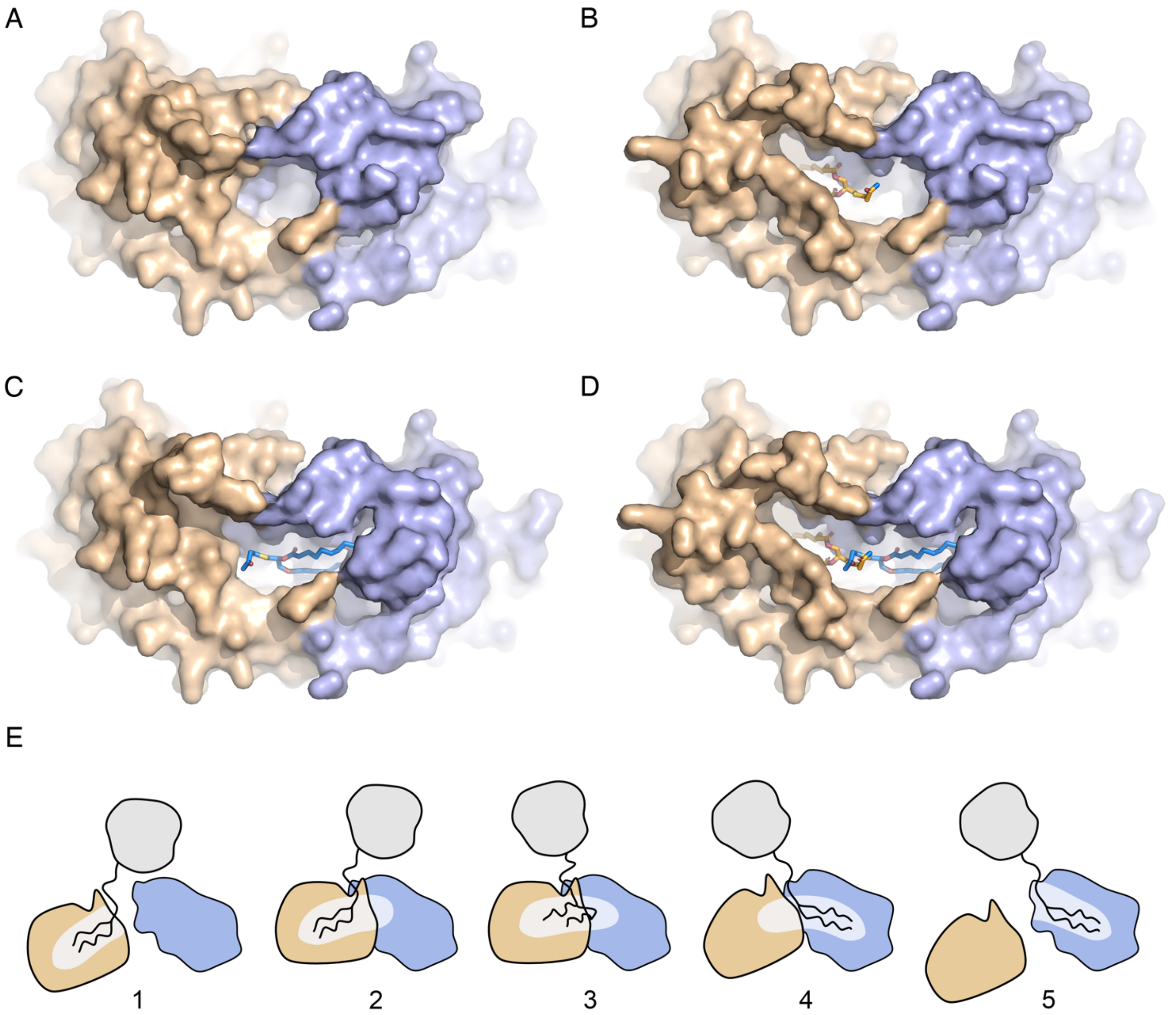

Supplementary Figure 9. Docking a lipid anchor to the LolA-LolB complex. (**A**) Closed state as observed in the lipid-free LolA-LolB complex where the pathways leading to the LolA (beige) and LolB (blue) cavities are both closed; (**B**) Lipid anchor modeled in LolA where the LolA lid is open to accommodate the lipid, and the path to the LolB cavity is closed; (**C**) Lipid anchor modeled in LolB where the LolB lid is open to accommodate the lipid, and the path to the LolA cavity is closed; (**D**) Overlay of the two states in (B) and (C) showing the predicted extent of the open complex during lipid transfer before the interactions between LolA and LolB have been fully broken. As discussed in the text, closed state in (A) is characterized by *i)* the helical lids in LolA and LolB being folded inwards (closed) on top of the concave β-sheets; *ii)* the LolB hook positioned to shield the opening to the LolA cavity; and *iii)* the indole ring in Trp81 in LolB oriented to prevent entry to the LolB cavity. (**E**) Proposed transfer steps. LolA has a diacyl lipid anchor bound in its open hydrophobic pocket and approaches LolB with a closed hydrophobic pocket (step 1). Formation of AB-specific interactions that are independent of the bound lipid accompanied with partial opening of the LolB hydrophobic pocket as the LolB hook docks with LolA (step 2). The interactions between LolA and LolB weakens gradually in response to gradual opening of the LolB cavity during initiation of LP transfer (step 3). Mostly complete transfer of lipid anchor to LolB and gradual closing of the LolA cavity as the LolA-LolB interactions break and the LolA lid starts to close (step 4). Complete transfer of LP to LolB and dissociation (step 5). The figure was prepared using PyMOL 2.4.2 (The PyMOL Molecular Graphics System, Version 2.0 Schrödinger, LLC).

Supplementary Table 1. Gene constructs, cloning primers and LIC vectors

Gene construct and cloning primers	LIC vector
The *Xcc lolA* gene construct codes for residues 21-209 in UniProt B0RT42. A periplasmic signal peptide corresponds to residues 1-21 and the mature protein starts at Gly22 in the translated genome sequence, which corresponds to Gly1 in the recombinant protein. The cloned gene construct starts at Ala21 which is the last amino acid of the periplasmic signal peptide and is therefore numbered 0 while the translation start Met is numbered -1.<br>Primers<br>Fwd: 5'-TTAAGAAGGAGATATACTATGGCAGGTGCGCGTC-3'<br>Rev: 5'-GATTGGAAGTAGAGGTTCTCTGCCTGTGCGTCGCCGACCAC-3'	pNIC-CTHO
The *Xcc lolB* gene construct codes for residues 22-218 in UniProt B0RUA2. A lipoprotein signal corresponds to residues 1-20 and the mature protein starts at Cys21 in the translated genome sequence. The cloned gene construct starts at Val22, which corresponds to Val2 in the recombinant protein. The translation start Met is numbered 1.<br>Primers<br>Fwd: 5'-TTAAGAAGGAGATATACTATGGTCTCAGTCCCGCGTGG-3'<br>Rev: 5'-GATTGGAAGTAGAGGTTCTCTGCTGGCGAGAGCGTCCATTG-3'	pNIC-CTHO

Supplementary Table 2. Data collection, phasing, and refinement statistics

**Data collection**[a]	*Xcc* LolA-LolB complex
Synchrotron, beamline	BioMAX, MAX IV
Wavelength (Å)	0.9763
Space group / mol per a.s.u.	R3:H / 2 LolA-LolB complexes
Cell dimensions: *a*, *b*, *c* (Å), α, β, γ (°)	137.51, 137.51, 145.21, 90.0, 90.0, 120.0
Resolution (Å), nominal	48.40–2.20 (2.30–2.20)
Unique reflections	51,938 (6,459)
R_{sym}	0.109 (2.325)
$I / \sigma I$	10.6 (1.3)
Completeness (%)	99.9 (99.8)
Redundancy	2.8 (2.3)
CC (1/2)[b]	99.8 (49.2)
Wilson *B* factor, isotropic (Å^2)	66.42
**Refinement**	
Resolution (Å)	48.4–2.20 (2.25–2.20)
Completeness	99.9
No. reflections work	51927 (5172)
No. reflections free	1995 (196)
R_{work}	0.220 (0.487)
R_{free}	0.260 (0.526)
N^o. non-hydrogen atoms: all, protein, ligands, water	5711, 5674, 10, 27
N^o. protein residues, all chains	730
Mean *B* factors: protein, ligand, water (Å^2)	75.2, 75.2, 88.4, 65.7
R.m.s.d bond lengths (Å), bond angles (°)	0.009, 1.09
Ramachandran: allowed, favored, outliers (%)	99.86, 96.23, 0.14
No. TLS groups	1
PDB ID	8ORN

[a] Statistics for outer-shell reflections are given in parentheses.

[b] Percentage of correlation between intensities from random half-datasets as given by XSCALE. Values given represent correlations significant at the 0.1% level (Karplus and Diederichs, 2012).

Supplementary Table 3. Hydrogen bonds and salt bridges that stabilize the *Xcc* LolA-LolB complex.

**LolA**	**LolB**	**Type**
Interactions present in both LolA-LolB heterodimers (AB-specific interactions)		
Gly185 N (β12A)	Arg52 O (β2B)	H-bond
Gly185 O (β12A)	Gly54 N (β2B)	H-bond
Ala187 N (β12A)	Gly54 O (β2B)	H-bond
Asp72 O (β5A/β6A)	Arg177 Nh1 (β10B)	H-bond
Asp72 O (β5A/β6A)	Arg177 Nh2 (β10B)	H-bond
Leu73 O (β5A/β6A)	Arg188 Ne (β11B), flexible	H-bond
Asp72 Od2 (β5A/β6A)	Arg45 Nh1 or NH2 (β1B)	Salt bridge
Asp182 Od2 (β12A)	Arg188 Nh1 (β11B)	Salt bridge
Interactions present only in $LolA_1$-$LolB_1$ (A/B interface)		
Gln75 Ne2 (β6A)	Ser49 Og (β1B)	H-bond
Asp149 Od2 (β10A)	Ser80 Og (β4B)	H-bond
Interactions present only in $LolA_2$-$LolB_2$ (C/D interface)		
Lys32 Nz (β2A)	Gln71 Oe1 (β3B)	H-bond

Supplementary Table 4. Hydrophobic residues in the LolA and LolB lipid-binding cavities[1]

Xcc LolA	*E. coli* LolA	*Xcc* LolB	*E. coli* LolB
Leu6	Leu6 (*sn-3* FA)	Leu34	Val25
Phe9 (*sn-1* FA)	(Arg9)	Trp40	Tyr31
Thr10	Leu10 (*sn-3* FA)	Phe42	(Thr33)
Leu13 (*sn-2* FA)	Val13 (*sn-1* FA)	Val46 (*sn*-1 FA)	Phe37
Leu16 (*sn-2* FA)	Phe16 (*sn-1* FA)	Ile48 (*sn*-1 FA)	Tyr39
(Gly18)	Ala18	Leu59 (*sn*-1 FA)	Phe50
Phe20	Phe20	Trp61 (*sn*-1 FA)	Trp52
Val24	Val24	Tyr68	Tyr60
Val39	Leu39 (*sn-1* FA)	Val70	Leu62
Leu41 (*sn-2* FA)	Val41 (*sn-1* FA)	Leu72 (*sn*-1 FA)	Leu64
Phe47 (*sn-2* FA)	Phe47 (*sn-2* FA)	Trp81 (*sn*-2 FA)	Leu73
Trp49 (*sn-1 sn-2* FA)	Trp49 (*sn-2* FA)	Leu83 (*sn*-2 FA)	Leu75
Tyr51	Met51	Leu94 (*sn*-2 FA)	Leu84
Ile59	Leu59 (*sn-2 sn-3* FA)	Leu97 (*sn*-2 FA)	(Asn87)
Val66	Leu66 (*sn-3* FA)	Val110	Met100
Val68	Phe68	Leu111 (*sn*-2 FA)	Ile101
(Gln81)	Leu81	Ala114	Leu104
(Glu84)	Ala84 (*sn-3* FA)	Trp117 (*sn*-1 FA)	Met107
Pro89 (*sn-1* FA)	Pro89 (*sn-3* FA)	Ile119 (*sn*-2 FA)	Ile109
Leu90 (*sn-2* FA)	Phe90 (*sn-2 sn-3* FA)	Pro120	Pro110
Leu93 (*sn-2* FA)	Ile93 (*sn-1 sn-2* FA)	Val121 (*sn*-2 FA)	Leu111
Ile94	Ala94	Met124 (*sn*-1 FA)	Leu114
Leu99	Asp100	Pro125	(Arg115)
Tyr103	Tyr104	Trp127	Trp117
Val105	Ile106	Val128	Ile118
Leu118	Phe113	Ala130	Gly120
Leu120	Leu115 (*sn-3* FA)	Pro149	Leu136
Phe130	Leu124	Val152	Ile139
Ala133	Phe127	Trp157	Trp148
Leu135 (*sn-1* FA)	Ile129 (*sn-3* FA)	Ile159	Val150
Phe137	Val131	Phe161	Tyr152
Leu142 (*sn-1* FA)	Ile137 (*sn-1* FA)	Trp164	Tyr155
Met145 (*sn-1* FA)	Phe140 (*sn-1 sn-3* FA)	Leu174	Met163
Ile157 (*sn-1* FA)	Tyr152 (*sn-1* FA)	Pro175	Pro164
Phe159 (*sn-1* FA)	Leu154 (*sn-1* FA)	Ile178	Met167
Trp162 (*sn-1* FA)	(Gln157)	Ala180 (*sn*-1 FA)	Leu169
		Val187 (*sn*-1 FA)	Ile176
		Leu189 (*sn*-1 FA)	Leu178
		Val191	Met180
		Trp194	Trp183
		Leu196	Val185

Accessible hydrophobic residues in the binding cavities of LolA and LolB. For the modeled diacyl lipid-bound states of *Xcc* LolA and LolB the interacting acyl chains are indicated in parenthesis for each residue, and the same applies to the hydrophobic cavity residues listed for the triacyl lipid-bound state of *E. coli* LolA (PDB 7Z6W; Kaplan et al., 2022). There is no experimental or theoretical structure available for *E. coli* LolB with a bound lipid anchor and therefore no acyl-chain interactions are listed. FA, fatty-acid chain.

Supplementary Table 5. Percent sequence identity

Protein sequence (UniProt)	% identity to *Xcc* LolA
*Xc*LolA (B0RT42)	Reference sequence
*Xf*LolA (Q9PDC7)	65.2
*Nm*LoLA (A9M2P9)	36.8
*Ng*LoLA (B4RJM6)	36.8
*Lp*LolA (Q5X4E7)	35.7
*Ps*LolA (Q9Z3U0)	33.9
*Pa*LolA (Q9I0M4)	31.7
*Yp*LolA (A0A5P8RWP4)	24.0
*Ec*LolA (P61316)	22.8
*Cv*LolA (Q9A261)	20.7
*Rp*LolA (Q9ZCA2)	17.8
Protein sequence	% identity to *Xcc* LolB
*Xc*LolB (B0RUA2)	Reference sequence
*Xf*LolB (Q9PA74)	51.0
*Pa*LolB (P42812)	30.3
*Ps*LolB (Q4ZXX0)	28.1
*Lp*LolB (CAH11756)	22.2
*Ec*LolB (P61320)	22.1
*Yp*LolB (A0A5P8RU70)	22.1
*Nm*LolB (A9M420)	18.2
*Ng*LolB (B4RKE9)	18.2
